# SOCS2-enhanced ubiquitination of SLC7A11 promotes ferroptosis and radiosensitization in hepatocellular carcinoma

**DOI:** 10.1038/s41418-022-01051-7

**Published:** 2022-08-22

**Authors:** Qianping Chen, Wang Zheng, Jian Guan, Hongxia Liu, Yao Dan, Lin Zhu, Yimeng Song, Yuchuan Zhou, Xinrui Zhao, Yuhong Zhang, Yang Bai, Yan Pan, Jianghong Zhang, Chunlin Shao

**Affiliations:** 1 Institute of Radiation Medicine, Shanghai Medical College, Fudan University, Shanghai, 200032 China; 2 Department of Radiation Oncology, Nanfang Hospital of Southern Medical University, Guangzhou, Guangdong 510515 China; 3 Department of Radiation Oncology, Shanghai Cancer Center, Shanghai Medical College, Fudan University, Shanghai, 200032 China

**Keywords:** Diagnostic markers, Tumour biomarkers

## Abstract

Radioresistance is a principal culprit for the failure of radiotherapy in hepatocellular carcinoma (HCC). Insights on the regulation genes of radioresistance and underlying mechanisms in HCC are awaiting for profound investigation. In this study, the suppressor of cytokine signaling 2 (SOCS2) were screened out by RNA-seq and bioinformatics analyses as a potential prognosis predictor of HCC radiotherapy and then were determined to promote radiosensitivity in HCC both in vivo or in vitro. Meanwhile, the measurements of ferroptosis negative regulatory proteins of solute carrier family 7 member 11 (SLC7A11) and glutathione peroxidase 4 (GPX4), intracellular lipid peroxidation and Fe^2+^ concentration suggested that a high level of ferroptosis contributed to the radiosensitization of HCC. Moreover, SOCS2 and SLC7A11 were expressed oppositely in HCC clinical tissues and tumour xenografts with different radiosensitivities. Mechanistically, the N-terminal domain of SLC7A11 was specifically recognized by the SH2-structural domain of SOCS2. While the L162 and C166 of SOCS2-BOX region could bind elongin B/C compound to co-form a SOCS2/elongin B/C complex to recruit ubiquitin molecules. Herein, SOCS2 served as a bridge to transfer the attached ubiquitin to SLC7A11 and promoted K48-linked polyubiquitination degradation of SLC7A11, which ultimately led to the onset of ferroptosis and radiosensitization of HCC. In conclusion, it was demonstrated for the first time that high-expressed SOCS2 was one of the biomarkers predicting radiosensitivity of HCC by advancing the ubiquitination degradation of SLC7A11 and promoting ferroptosis, which indicates that targeting SOCS2 may enhance the efficiency of HCC radiotherapy and improve the prognosis of patients.

## Introduction

Hepatocellular carcinoma (HCC) is one of the most prevalent malignancies worldwide [1–3]. Due to the insidious onset of HCC and the absence of early pathological symptoms, most patients are diagnosed as advanced when discovered and exhibit high resistance to chemotherapy and low success rate of radical surgery [4–6]. Since HCC cells are moderately sensitive to radiation, equivalent to the radiosensitivity of hypofractionated squamous cell carcinoma [7], progressive HCC patients are often treated with radiotherapy [8, 9]. Whereas in fact, the endogenous and therapy-induced radioresistance limits the efficacy of HCC radiotherapy [10–12]. Hence, the potential targets or molecular mechanisms engaged in HCC radioresistance must be appropriately understood to avoid radiotherapy failure.

The suppressor of cytokine signaling (SOCS) family, with eight members including cytokine-inducible SH2 protein (CIS) and SOCS 1–7 proteins [13], has been characterized as major regulators of cytokine signaling factors since they were discovered in 1999 [14]. SOCS proteins consist of analogous structural components, a conserved C-terminus domain (CTD) including a SOCS-BOX region, a central SH2-structural domain, and an N-terminal domain (NTD) of variable length and sequence [15, 16]. Among these domains, SOCS-BOX region of SOCS1 and SOCS6 can form a stable complex with elongin B/C and assemble into an E3 ubiquitin ligase complex to exert ubiquitination [17, 18]. Additionally, imbalance of SOCS2 was involved in the occurrence, development, metastasis and prognosis of HCC [19, 20]. However, no investigation has been conducted to elucidate the relationship between SOCS2 and radiosensitivity in either HCC or other tumors. Mechanistically, previous literatures hold an opinion that interference with SOCS2 could partially mediate apoptosis or necrosis, ultimately impact the progression of HCC cells. However, further investigation is still required to know whether the activation of SOCS2 can be involved in other forms of regulated cell death (RCD) such as ferroptosis.

Ferroptosis is a newly discovered and iron-dependent RCD that differs from apoptosis, necroptosis and autophagy morphologically, genetically and biochemically [21, 22]. The occurrence of ferroptosis is characterized by the accumulation of cytotoxic lipid peroxides [23–25]. Recently, direct induction or secondary regulation through targeting ferroptosis-associated proteins has emerged as an efficacious therapeutic approach to trigger cancer cell death, especially to radioresistant malignancies [26]. It was reported that ferroptosis and its regulatory proteins, such as glutathione peroxidase 4 (GPX4), solute carrier family 7 member 11 (SLC7A11) and P53 played crucial roles in hepatocarcinoma [27–29]. Nevertheless, it remains obscure whether other potential proteins could regulate ferroptosis and subsequently influence the prognosis of HCC, especially the tolerance and recurrence of HCC after radiotherapy.

In previous study, we have constructed radioresistant HCC cells to identify the potential proteins involved in HCC radioresistance using RNA-seq analysis. Herein, we identified that SOCS2 could specifically expedite the ubiquitination degradation of SLC7A11 and thus contributed to ferroptosis and radiosensitization of HCC, indicating that targeting SOCS2 may represent a promising therapeutic strategy for HCC radiotherapy.

## Materials and methods

### Cell culture

Human HCC cell lines SK-Hep-1 and HepG2 were purchased from Shanghai Cell Bank (Chinese Academy of Science, Shanghai, China). HCC radioresistant cell lines SK-Hep-1R and HepG2R was constructed from SK-Hep-1 and HepG2 cells by irradiating with 8 Gy for 5 cycles and 10 Gy for 2 additional cycles (8, 8, 8, 8, 8, 10, 10 Gy) of X-rays at a dose rate of 1 Gy/min. Cells were cultured in DMEM medium (Gibco, Hangzhou, China) supplemented with 10% fetal bovine serum (Gibco, NY, USA) and 100 U/ml penicillin and 100 μg/ml streptomycin (YEASEN Biotech Co. Ltd., Shanghai, China) in a humidified atmosphere of 5% CO_2_ and 95% air. These cells were used till passage 20. All cell lines authenticated by Short Tandem Repeat (STR) assay were mycoplasma free.

### Patient samples

Twenty-four HCC specimens were collected from the patients who underwent hepatectomy or ultrasonically guided liver biopsy before radiotherapy in the Nanfang Hospital of Southern Medical University of China. The detailed clinical information of patients was shown in Supplementary Table S1. The progression and prognosis of these HCC patients were evaluated by CT before and after radiotherapy according to the Response Evaluation Criteria in Solid Tumor 1.1 (RECIST 1.1) criteria. Radioresistant HCC patients (12 specimens) were defined as ones with recurrence in liver, lung, skeleton, lymph and other positions after irradiation (IR). Conversely, radiosensitive HCC patients (12 specimens) hold good progression without recurrence.

For clinical tissue-related experiments, all studies were carried out in accordance with international guidelines and ethical standards of the World Medical Association (Declaration of Helsinki). All subjects gave written informed consent in advance. For the experiments using human participants or data, prior approval was obtained from the Nanfang Hospital of Southern Medical University Institutional Board (Guangzhou, China).

### Xenograft tumor mouse model

Nude mice (6 weeks, male, BALB/C-nu/nu, SIPPR/BK Lab. Animal Co. Ltd., Shanghai, China) were selected randomly and applied for ectopic tumor construction. Since HepG2 and HepG2R cells are non-tumorigenicity according to ATCC (https://www.atcc.org/), we just injected SK-Hep-1, SK-Hep-1R cells, and SK-Hep-1R cells transfected with lentiviral vector carrying human *SOCS2* gene (lvSOCS2) (SK-Hep-1R-lvSOCS2) or its negative control cells (SK-Hep-1R-lvNC) (1 × 10^7^/100 μL) into the left flank of nude mice. These mice were divided into 8 groups (*n* = 6/group) according to the type of inoculated cells and IR or not. When tumor size reached about 100 mm^3^, IR groups were treated with 8 Gy X-rays for 3 consecutive days. Only tumor locations were exposed to IR, and the other mouse body was shielded with a lead plate. Then tumor volume (V) was measured every 3 days using the standard formula V  =  L*W^2^/2 (L = length, W = width). When the volume approached to 1000 mm^3^, the mice were sacrificed by cervical dislocation and the xenograft tumors were excised, photographed, and cut into two parts where one half was fixed and embedded in paraffin for immunofluorescence staining, and the remaining half was flash-frozen in liquid nitrogen for reserve.

For nude mice-related experiments, all studies complied with the Animal Research: Reporting of In Vivo Experiments (ARRIVE) guidelines and carried out in accordance with the National Institutes of Health guide for the care and use of Laboratory animals (NIH Publications No. 8023, revised 1978). The animal experimental protocol was approved by the Animal Welfare and Ethics Committee of Fudan University (20171304A215). Furthermore, there were no blinding study for different animal groups.

### RNA Isolation and sequencing analysis

Total cellular RNA was extracted using TRIzol reagent (Invitrogen, San Diego, CA, USA) following manufacturer’s recommendation. RNA-sequencing (RNA-seq) analysis was performed on an Illumina Hiseq 2500 platform and its library was prepared using rRNA-depleted RNA by NEBNext^®^ Ultra™ Directional RNA Library Prep Kit for Illumina^®^ (NEB, Ipswich, MA, USA) in a 20 ng-RNA reaction system. The differentially expressed genes (DEGs) was derived from various cell samples (SK-Hep-1R and SK-Hep-1). Transcripts with *p*-value < 0.05 were assigned as differentially expressed mRNAs (DEmRNAs). All sequencing programs and analyses were performed by Novogene Company (Beijing, China).

### Databases description

#### HCCDB database analysis

Hepatocellular Carcinoma Expression Atlas Database (HCCDB) database (http://lifeome.net/database/hccdb/home.html, accessed 1st Sep, 2021) is a database of hepatocellular carcinoma expression atlas containing 12 public HCC gene expression datasets in total 3917 samples, including the data from Cancer Genome Atlas (TCGA), Gene Expression Omnibus (GEO) and Liver Cancer - RIKEN and JP Project from International Cancer Genome Consortium (ICGC LIRI-JP). HCCDB provides the visualization for the results from several computational analyses, such as DEGs analysis, tissue-specific and tumor-specific expression analysis.

#### UALCAN database analysis

UALCAN database (http://ualcan.path.uab.edu, accessed 1st Sep, 2021) is a cancer microarray database and web-based data-mining platform using TCGA level 3 RNA-seq and clinical data from 31 cancer types. UALCAN was applied to investigate the association between SOCS2 expression and cancer stages of HCC.

#### GEPIA database analysis

Gene Expression Profiling Interactive Analysis (GEPIA) database (http://gepia.cancer-pku.cn/index.html, accessed 1st Sep, 2021) is a user-friendly and interactive web portal for gene expression analysis based on TCGA and GTEx data including 9736 tumors and 8587 normal samples. In the current study, GEPIA was allied to investigate the SOCS2 expression across tumor and normal samples, as well as the generation of survival curves based on gene expression with the log-rank test and the Mantel-Cox test in HCC.

### Immunofluorescence (IF) and immunohistochemistry (IHC) assay

IF assay: HCC samples were fixed in formalin and embedded in paraffin and then cut into 4-μm thick tissue slices. The tissue slices from each group were incubated with anti-mouse SOCS2, anti-mouse GPX4, and anti-rabbit SLC7A11 antibodies at 4 °C overnight. Then these slices were rinsed with PBS and incubated with secondary antibody for 2 h at 37 °C, followed by treatment with DAPI (Beyotime Biotechnology, Haimen, China) for 10 min. The secondary antibodies included goat anti‐mouse IgG FITC, goat anti‐rabbit IgG Cy3 and goat anti‐mouse IgG Cy5. The tissue images of at least 3 fields were randomly captured using a fluorescence microscope (Nikon Eclipse CI-S, Tokyo, Japan) and analyzed with the Image J software (Version 1.8.0.112, National Institutes of Health, USA). The detail information of relevant antibodies was listed in Supplementary Table S2.

IHC assay: The xenograft tumors were fixed overnight in 10% neutral buffered formalin and then transferred to 70% ethanol, embedded and sectioned. Next, the tumor masses were stained using anti-rabbit-4-HNE, anti-rabbit-SOCS2, anti-rabbit SLC7A11 and anti-mouse-GPX4 antibodies and counterstained using hematoxylin. IHC images were obtained using a microscope at 400× magnification. IHC staining was analyzed semi-quantitatively from three independent, random fields using Image J software. The analysis index was the average optical density (AOD; AOD = Integrated optical density (IOD)/Area). The detail information of relevant antibodies was listed in Supplementary Table S2.

### Cell and tumor irradiation

HCC cells were irradiated at various doses with an X-ray irradiator (X‐RAD 320, Precision X-Ray, Inc., North Branford, CT; 12 mA, 2‐mm aluminum filtration, USA). For in vivo tumor IR, nude mice with HCC xenograft were anesthetized with ketamine/xylazine (100 mg/kg + 10 mg/kg) and placed in a customized lead mould to accept local IR of X-rays.

### Colony formation assay

The radiosensitivity of HCC cells was determined using a colony formation assay. Cells (100–1200/well) were seeded into 6-well plates and treated with different doses of IR (0, 2, 4, 6 Gy) in triplicate. After IR, cells were cultured for 9–12 days to form colonies and then fixed and stained with crystal violet. The colonies (with > 50 cells) were counted for the survival fraction calculation, and the survival curves were fitted with the formula SF  =  1-(1-exp(−k*D))ˆN based on the single-hit multi-targeted model.

### Western blot (WB) analysis

Total protein of HCC cells was extracted with RIPA lysate buffer (Beyotime Biotechnology) according to the manufacturer’s instruction. After measuring the protein concentrations by BCA protein assay, 40 μg of total protein was loaded and separated by 10% SDS-PAGE gel, transferred on polyvinylidene fluoride (PVDF) membranes (Immobilon-P; Millipore Corporation, MA, USA), blocked with 5% skim milk diluted by 0.05% Tris-buffered saline/Tween (TBST), and incubated with primary antibodies overnight at 4 °C, then incubated with horseradish peroxidase-linked IgG secondary antibody (Beyotime Biotechnology) at room temperature for 2 h. The protein bands were exposed with enhanced chemiluminescence (ECL) kit (Millipore, St. Louis, MO, USA) following the intensity analysis with the Bio-Rad ChemiDoc XRS system (Bio-Rad, Hercules, CA, USA). The detail information of relevant antibodies was listed in Supplementary Table S2.

### Measurement of intracellular Fe^2+^ content

Cells were seeded at a density of 1 × 10^5^ cells/ml in a 12-well culture plate. The level of intracellular Fe^2+^ ions was measured with a Ferro-orange kit (Dojingo, Molecular Technologies Inc., Shanghai, China) according to the manufacturer’s instruction. Briefly, cells were irradiated with 4 Gy of X-rays, incubated in serum-free medium for 4 h, and stained with 1 μM Ferro-orange in HBSS for 30 min at 37 °C. Then the fluorescence absorbance of culture was immediately detected in an automatic microplate spectrophotometer (Synergy H1, BioTek Instruments, Vermont, USA) with an excitation wavelength 543 nm and an emission wavelength 580 nm.

### Measurement of intracellular lipid peroxidation

To visualize intracellular lipid peroxidation, HCC cells in logarithmic growth were irradiated with 4 Gy of X-rays, cultured for 4 h in serum-free medium, stained with 10 μM Liperfluo (Dojindo Molecular Technologies Inc.) for 30 min, washed, imaged by a high-content imaging system (ImageXpress Micro 4, Molecular Devices, San Francisco, CA, United States), and analyzed with the ImageJ software.

### Real-time quantitative PCR

RNA Kit I (Omega, Norcross, GA, USA) was used to isolate RNA from cells. According to the manufacturer’s protocol, reverse transcription of total RNA to cDNA was carried out in 20 μl reaction reagents of the qRT-PCR Kit (Tiangen, Beijing, China). *ACTB* was used as an internal control. For *SOCS2* gene, the forward primer was 5′-TTA AAA GAG GCA CCA GAA GGA AC-3′ and the reverse primer was 5′- AGT CGA TCA GAT GAA CCA CAC T-3′. For *β-Actin* gene, the forward primer was 5′-CAT GTA CGT TGC TAT CCA GGC-3′ and the reverse primer was 5′- CTC CTT AAT GTC ACG CAC GAT-3′. For *SLC7A11* gene, the forward primer was 5′- TCT CCA AAG GAG GTT ACC TGC-3′ and the reverse primer was 5′- AGA CTC CCC TCA GTA AAG TGA C-3′.

### Small interfering RNA (siRNA) transfection

According to the manufacturer’s instruction, HCC cells were respectively transfected with the *SOCS2*-specific siRNA, *elongin B* specific siRNA and *elongin C* specific siRNA at a final concentration of 100 nM using riboFECTTM CP Transfection Agent (RiboBio CO., Ltd, Guangzhou, China). A scrambled siRNA was applied as a negative control. The transfection efficiency was evaluated by Western blot assay at 48-72 h after transfection. The sequences of siRNAs were as follows. si*SOCS2*: 5′- GAA GGA ACT TTC TTG ATTA-3′; si*elongin B*: 5′-GGG AAG CAG UGC CAA UGA AdTdT-3′; si*elongin C*: 5′-AAG AGA ACA UGC AUU AAC AdTdT-3′; siNC: 5′-TTC TCC GAA CGT GTC ACGT-3′.

### Plasmid and transfection

Using full-length SOCS2 and SLC7A11 amplicons as templates, a series of SOCS2-truncated (SOCS2 amino acids 1–40, 40–156 and156–198), SOCS2-ΔSH2, SLC7A11-truncated fragments (SLC7A11 amino acids 1–43, 1–470 and 470–501) and SLC7A11-ΔNTD was amplified by PCR and cloned into Flag-tagged destination vectors (Genechem, Shanghai, China). The mutant SOCS2 plasmid was also designed by Genechem Technology. Additionally, HA-ubiquitin plasmid and its mutant (K48R, K63R) plasmid were purchased from Hanyin Biotechnology (Shanghai, China) by cloning the corresponding human full-length DNA sequence into the HA-pcDNA 3.1(+). All transfection experiments applied Lipofectamine 3000 reagents (L3000015, Invitrogen, Eugene, OR, USA) according to the manufacturer’s protocols.

### Transfection of lentiviral vector

Lentiviral vectors (Ubi-CMV-SOCS2-SV40-puro) were purchased from Genechem (Shanghai, China). The empty vector was used as the negative control. HCC cells were cultured with lentivirus for SOCS2 transfection and the antibiotic-resistant transfected cells were selected and enriched by treatment of puromycin (Hanyin Biotechnology) at a final concentration of 2 μg/ml in culture medium. The efficiency of overexpressed-SOCS2 in HCC cells was monitored by Western blot assay.

### Co-immunoprecipitation (Co-IP) assay

According to the manufacturer’s instruction, the whole cell lysates (WCL) were collected and centrifuged at 10,000 *g* for 10 min at 4 °C (Beyotime Biotechnology). Then 1 ml supernatant was incubated with 1 μg anti-SOCS2 antibody (mouse/rabbit), anti-SLC7A11 antibody (rabbit), anti-Flag antibody (mouse) and anti-IgG antibody (mouse/rabbit) for 16 h followed by addition of 20 μl fresh protein A/G plus agarose beads (Santa Cruz Biotechnology, Shanghai, China) and incubated overnight at 4 °C. Samples were spun down, washed four times with immunoprecipitation buffer and fractionated by SDS-PAGE followed by Western blot analysis. Relevant antibodies are detailed in Supplementary Table S2.

### Ubiquitylation assay

HCC cells were treated with 10 μM MG132 (MedChemExpress, Shanghai, China) and then exposed to IR. After 4 h, these cells were washed with PBS and lysed for immunoprecipitation assay using 1 μg anti-SLC7A11 antibody followed by Western blot assay using anti-HA or anti-Ub to detect SLC7A11 ubiquitination.

### Drug treatments

HCC cells were treated with 0.2 μM of 1 S,3R-RSL3 (RSL-3) for 4 h to induce ferropotosis, treated with 1 μM of harringtonine (HT) for 2-12 h to inhibit protein synthesis, treated with 10 μM of MG132 for 4 h to inhibit proteasome activity, or treated with 50 μM of leupeptin for 4 h to inhibit the lysosomal degradation. All of these drugs were purchased from MedChemExpress (Shanghai, China). Besides, HCC cells were treated with 5 μM Ferrostatin-1 (Fer-1) to inhibit lipid peroxidation (Abclonal Technology, Wuhan, China).

### STR identification

STR identification was performed by Chinese Academy of Science (Shanghai, China) and the results for HepG2 and SK-Hep-1 were as follows: HepG2, D5S818: 11,12; D13S317: 9,13; D7S820: 10,10; D16S539: 12,12; vWA: 17,17; THO1: 9,9; Amelogenin: X, Y. TPOX: 8,9; CSF1PO: 10,11; SK-Hep-1, D5S818: 10,13; D13S317: 8,12; D7S820: 8,11,12; D16S539: 12,12; vWA: 14,17; THO1: 7,9; Amelogenin: X, X; TPOX: 9,9. CSF1PO: 11,12. These results were consistent with the DNA profiles reported by ATCC and DSMZ, indicating no other human cell contamination in these cells.

### Statistical analysis

SPSS 19.0 and GraphPad Prism software (GraphPad Software, Inc.) were used to perform statistical analyses. Student’s *t*-test or ANOVA was used for group comparison. Pearson’s correlation coefficient was used to assess the relationship between SLC7A11 and SOCS2 expression in clinical samples and transplanted tumors. Overall survival curves were plotted via the Kaplan–Meier method and compared by the log-rank test. Bars and errors represent the mean ± standard deviation (SD) of repeated measurements; *P* < 0.05 was considered significant. Unless otherwise stated, experiments were performed independently in triplicate with the sample number “*n*” indicated in the figure legend. All gel images of the relative protein expression were analyzed by Quantity one software (version 4.6.2). The relative expression levels of all proteins in immunofluorescence and immunohistochemistry were analyzed by Image J software.

## Results

### SOCS2 negatively correlated with radiosensitivity of HCC

**Fig. 1:**
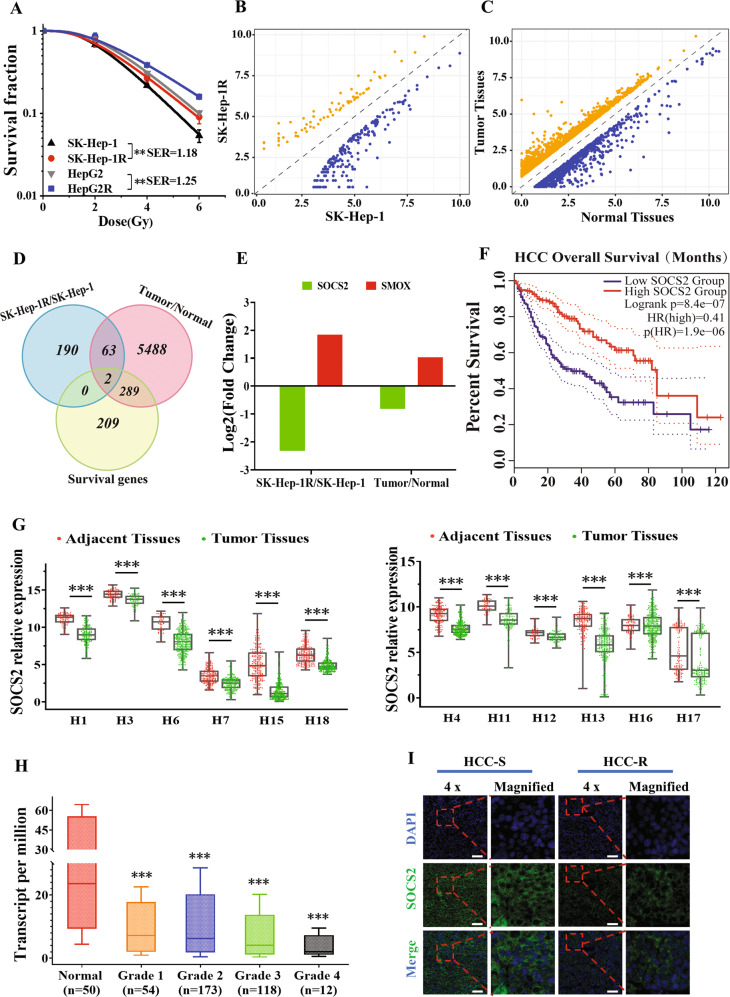
Overexpression of SOCS2 was correlated with radiosensitization of hepatocellular carcinoma. **A** Dose responses of survival fractions of SK-Hep-1, SK-Hep-1R, HepG2 and HepG2R after IR. **B** Symmetric scatter diagram of differentially expressed genes (DEGs) between SK-Hep-1R and SK-Hep-1 cells. **C** Symmetric scatter diagram of DEGs between the HCC tumor and paracancerous tissues from TCGA and GEPIA dataset (download data: 2021-09-01). **D** Venn diagram of the co-expressed DEGs among above two groups (SK-Hep-1R/SK-Hep-1, tumor/normal tissues) and the survival genes for prognosis prediction of HCC from TCGA and GEPIA dataset. **E** Fold changes of SOCS2 and SMOX gene expressions in SK-Hep-1R cells and tumor tissues in comparison with SK-Hep-1 cells and normal tissues, respectively. **F** Kaplan-Meier curves of HCC survivals based on the expression status of SOCS2 gene according to TCGA and GEPIA dataset. **G** Box scatter diagrams of the relative expression level of SOCS2 in tumor and adjacent normal tissues according to 12 cohorts in HCCDB. The central mark is the median; the edges of the box are the 25th and 75th percentiles. H refers to HCCDB. **H** Boxplot of the relative expression level of SOCS2 in normal and tumor tissues of HCC patients with four pathological grade 1, 2, 3 or 4 from UALCAN database. The central mark is the median; the edges of the box are the 25th and 75th percentiles. **I** Representative immunofluorescence images of SOCS2 protein in tumor tissues of radioresistant HCC patients (HCC-R) (*n* = 12) and radiosensitive HCC patients (HCC-S) (*n* = 12). Nuclei were stained with DAPI (x4). Scale bars, 100 μm. *P < 0.05, **P < 0.01 and ***P < 0.001 between indicated groups.

After long-term fractional IR with a total dose of 60 Gy, two radioresistant HCC cell lines SK-Hep-1R and HepG2R were constructed from their parental cell lines SK-Hep-1 and HepG2 with a sensitivity enhancement ratio (SER) of 1.18 and 1.25, respectively (Fig. 1A). To determine potential genes involved in HCC radioresistance, differential mRNA profiles between SK-Hep-1 and SK-Hep-1R cells were explored by RNA-seq analysis, and a total of 255 mRNAs were identified. Among them, 189 mRNAs were upregulated while 66 mRNAs were downregulated (Supplementary Table S3), shown as symmetric scatter diagrams in Fig. 1B. Moreover, the analysis of GEPIA and TCGA database identified that 5856 genes were differentially expressed in HCC tissues in comparison with normal liver tissues (Fig. 1C), including 4282 upregulated genes and 1572 downregulated genes (Supplementary Table S4). We then combined above differentially expressed genes (DEGs) with the survival genes related to HCC prognosis based on GEPIA database (Supplementary Table S5) to search for DEGs regulating radioresistance and prognosis of HCC. Venny analysis revealed that only two genes, *SOCS2* and *SMOX*, were downregulated or upregulated simultaneously in these datasets (Fig. 1D). Particularly, *SOCS2* gene was obviously decreased in HCC tissues and radioresistant SK-Hep-1R cells compared with normal tissues and SK-Hep-1 cells (Fig. 1E), while a low expression of *SOCS2* referred to a poor prognosis of HCC patients according to the Kaplan–Meier overall survival (OS) analysis with GEPIA (Fig. 1F). Besides, *SMOX* gene was increased in HCC tissues and SK-Hep-1R cells compared with normal tissues and SK-Hep-1 cells (Fig. 1E), and the advanced *SMOX* indicated a poor prognosis of HCC (Fig. S1A).

To further certificate the role of these two genes in the occurrence and progression of HCC, we applied HCCDB database for further analysis. Figure 1G revealed that the mRNA expression of *SOCS2* in HCC tissues was significantly lower than that in adjacent normal tissues from 12 HCCDB cohorts, suggesting that high expression of *SOCS2* inhibited HCC occurrence and progression. However, *SMOX* was downregulated in cohort 6 and 11 but upregulated in other cohorts (Fig. S1B), whose consistency was worse than *SOCS2* in HCC regulation. Therefore, *SOCS2* was of higher significance as a potential gene involved in the radioresistance of HCC. Interestingly, patients with higher *SOCS2* expression also had a significantly longer OS in HCCDB6 and HCCDB15 (Fig. S1C, D). Moreover, compared with 50 normal hepatic samples, *SOCS2* in HCC stage IV was lower than that in stage I, II and III (Fig. 1H), which was in consistent with the phenomenon that patient of low *SOCS2* expression possessed a poor prognosis. To determine whether SOCS2 directly mediated the prognosis of HCC after radiotherapy, we randomly selected appropriate tumor tissues from 12 radiosensitive patients and 12 radioresistant patients of HCC for immunofluorescence, and found that radioresistant tissues exhibited lower SOCS2 expression (Fig. 1I; Fig. S1E). To sum up, these results imply that SOCS2 may act as an anti-oncogene to suppress HCC progression and radioresistance.

### Overexpression of SOCS2 increased radiosensitivity of HCC in vitro and vivo

**Fig. 2:**
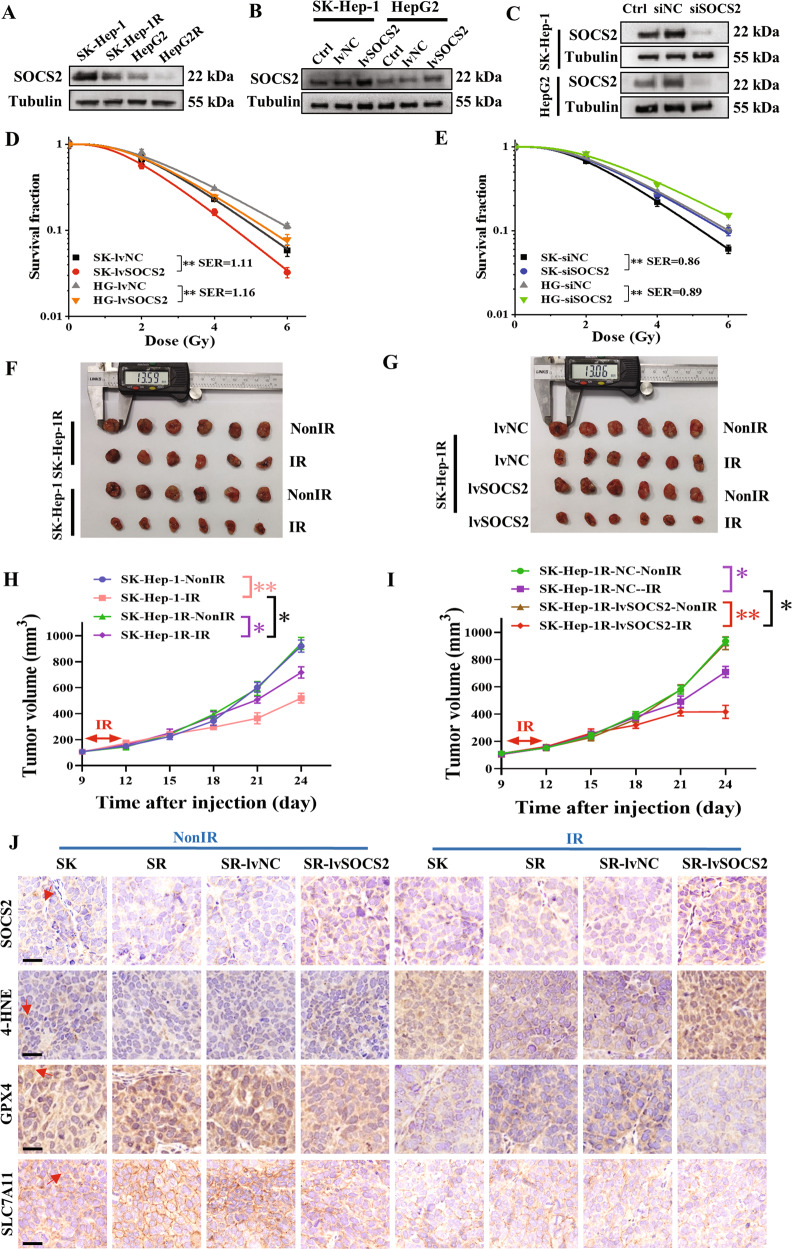
SOCS2 overexpression sensitized HCC to IR (IR) in vivo and in vitro. **A** Western blot assay of SOCS2 and tubulin proteins in SK-Hep-1, SK-Hep-1R, HepG2 and HepG2R cells. **B**, **C** Western blot assay of SOCS2 and tubulin proteins in SK-Hep-1 and HepG2 cells transfected with lvSOCS2, *siSOCS2* or their negative control (lvNC, siNC). **D** Dose responses of survival factions of SK-Hep-1 and HepG2 cells with or without lvSOCS2 transfection. SK refers to SK-Hep-1, HG refers to HepG2. **E** Dose responses of survival factions of SK-Hep-1 and HepG2 cells with or without *siSOCS2* transfection. **F**, **G** General view of tumor mass of each indicated group at 24 days after cell injection. **H**, **I** Tumor volume of above groups was examined every 3 days until 24 days after subcutaneously cell injection. **J** Representative immunohistochemistry images of the expressions of SOCS2, 4-HNE, GPX4 and SLC7A11 protein in the aforementioned xenograft tumors (x40). The red arrow indicated the spot or region where positive protein expression was present. Scale bars, 20 μm. *P < 0.05, **P < 0.01 and ***P < 0.001 between indicated groups.

We then detected the relationship of SOCS2 with radiosensitivity of HCC cells. Western blot assay illustrated that SOCS2 decreased orderly in SK-Hep-1, SK-Hep-1R, HepG2 and HepG2R cells (Fig. 2A, Fig. S2A), consistent with their radiosensitivity (Fig. 1A). To clarify the function of SOCS2, HCC cells were transfected with lvSOCS2 or si*SOCS2* to effectively enhance or reduce SOCS2 expression (Fig. 2B, C; Fig. S2B, C). Overexpressed SOCS2 caused a remarkable reduction in survival fraction of SK-Hep-1 and HepG2 cells after IR with a SER of 1.11 and 1.16, respectively (Fig. 2D). Conversely, si*SOCS2* increased the radioresistance of HCC cells with a SER of 0.86 and 0.89 (Fig. 2E).

Next, we further examined whether the same phenomenon could be observed in vivo. We found that IR elicited a reduction in the volume of xenografts, more pronounced in SK-Hep-1 than in SK-Hep-1R cells (Fig. 2F, H; Fig. S2D), and the overexpression of SOCS2 caused a further reduction in xenograft volume post-IR (Fig. 2G, I; Fig. S2D).

Since radiation-induced ferroptosis has been well determined by Gan and Zou et al. [30, 31], we wondered whether ferroptosis would function in the radiosensitivity of tumors. Given that the doubling time of SK-Hep-1 and SK-Hep-1R cells was approximately 24 h, we examined the expressions of ferroptosis-related proteins in xenografts at 12 h and 24 h post-IR by western blotting assay and found an increase of SOCS2 and a reduction of GPX4, with more significant changes at 12 h. (Fig. S2E). Moreover, IHC assay was performed to detect the expressions of SOCS2 and ferroptosis proteins in xenografts unirradiated or at 12 h post-IR. It was found that IR increased the expressions of SOCS2 and ferroptosis marker (4-HNE) but inhibited GPX4 and SLC7A11 expressions (Fig. 2J; Fig. S2F). Besides, overexpression of SOCS2 reduced GPX4 or SLC7A11 but promoted 4-HNE in xenografts. Collectively, SOCS2 promoted radiosensitization of HCC probably by inducing ferroptosis.

### Ferroptosis contributed to the radiosensitization of HCC tissues and cells

**Fig. 3:**
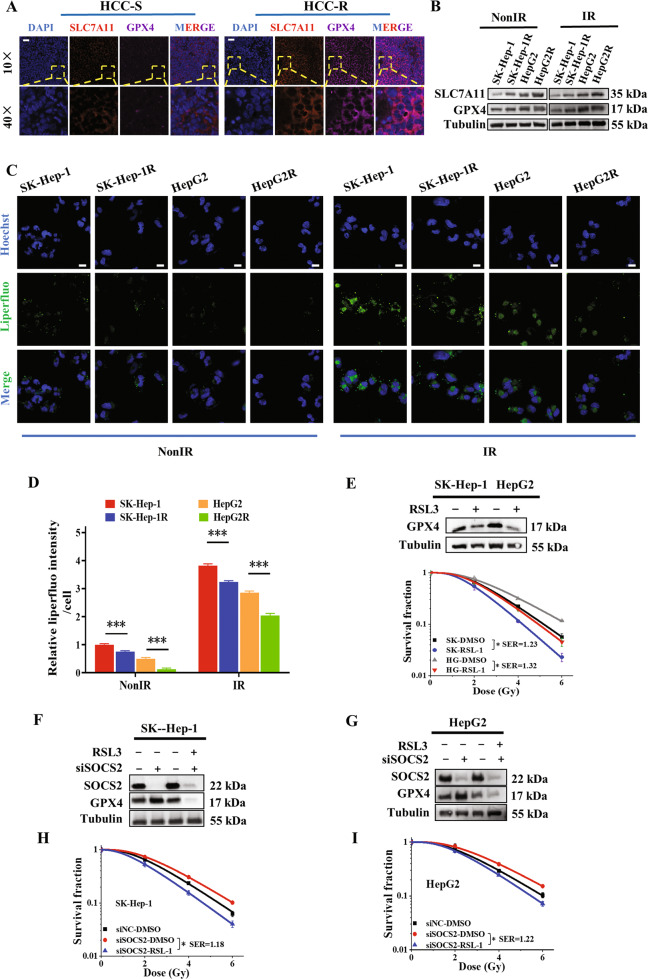
Enhanced ferroptosis resulted in radiosensitization of HCC. **A** Representative fluorescence images of SLC7A11 and GPX4 proteins assessed by immunofluorescence assay in HCC clinical tissues from 12 radioresistant (HCC-R) and 12 radiosensitive (HCC-S) patients. Nuclei were stained with DAPI (×10, ×40). Scale bars, 50 μm. **B** Western blot assay of SLC7A11, GPX4 and tubulin proteins in SK-Hep-1, SK-Hep-1R, HepG2 and HepG2R cells at 4 h after 4 Gy IR or non-IR. Representative images (**C**) and quantification (**D**) of the relative fluorescence intensity of liperfluo (a lipid peroxide fluorescent probe) in SK-Hep-1, SK-Hep-1R, HepG2 and HepG2R cells at 4 h after 4 Gy IR or non-IR. Nuclei were stained with Hoechst (×40). Scale bars, 10 μm. **E** Dose responses of survival factions of SK-Hep-1 and HepG2 cells treated with RSL3, and the western blot assay of GPX4 and tubulin proteins in these cells. SK refers to SK-Hep-1, HG refers to HepG2. **F**, **G** Western blot assay of GPX4 and tubulin proteins in HCC cells treated with RSL3, *siSOCS2* or its control siNC. **H**, **I** Dose responses of survival factions of SK-Hep-1 and HepG2 cells treated with RSL3, *siSOCS*2 or its control siNC. *P < 0.05, **P < 0.01 and ***P < 0.001 between indicated groups.

To further verify above conjecture, we examined the expressions of GPX4 and SLC7A11 in HCC clinical tissues and found that radiosensitive tissues had a higher level of ferroptosis (Figs. 3A; S3A). We then evaluated the level of ferroptosis in HCC cells. It was found that GPX4 and SLC7A11 also increased along with cell radioresistance with IR or not (Fig. 3B, Fig. S3B). Next, results of the fluorescent probe-liperfluo [32] revealed that the hydroperoxide lipid levels increased with HCC radiosensitivity, rising higher after IR (Fig. 3C, D), whereas the lipid peroxidation inhibitor (Fer-1) would interfere peroxide-lipid production (Fig. S3C, D). Since iron overload is another major character of ferroptosis, the level of cytosolic Fe^2+^ content was also measured by Ferro-Orange probes in HCC cells. Outcomes showed that Fe^2+^ content increased with higher radiosensitivity and it was augmented after IR (Fig. S3E). These results implied that radioresistant HCC cells possessed lower level of ferroptosis.

To further scrutinize whether ferroptosis conducted the HCC radiosensitization, we promoted the production of ferroptosis using RSL3 (an inhibitor of GPX4) and found an increment in radiosensitivity of SK-Hep-1 and HepG2 cells (Fig. 3E; Fig. S3F). We then treated si*SOCS2*-transfected HCC cells with RSL3 and found the decline of GPX4 expression and radiosensitization in cells (Fig. 3F, G, H, I; Fig. S3G, H). Taken together, ferroptosis contributed to radiosensitization of hepatoma cells, and the acquired radioresistance owing to SOCS2-suppressed could be reversed by RSL3-induced ferroptosis.

### SOCS2 positively related to ferroptosis

**Fig. 4:**
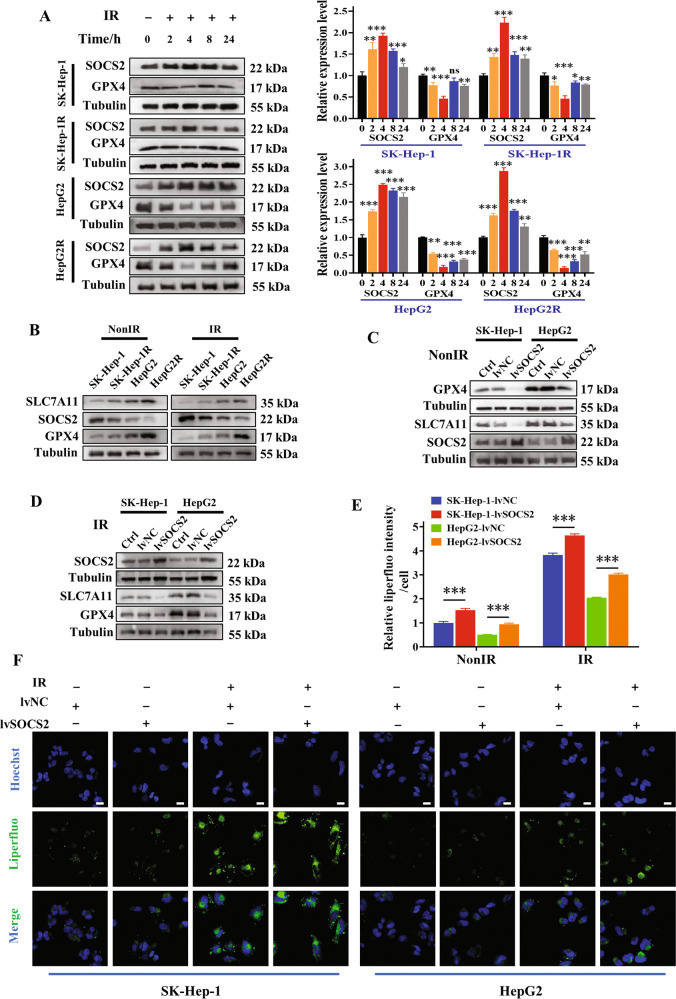
SOCS2 facilitated ferroptosis in HCC cells. **A** Western blot assay of GPX4 and SOCS2 proteins and their relative levels in SK-Hep-1, SK-Hep-1R, HepG2 and HepG2R cells at 2, 4, 8, 24 h after 4 Gy IR or non-IR. **B** Western blot assay of SLC7A11, GPX4 and SOCS2 proteins in SK-Hep-1, SK-Hep-1R, HepG2 and HepG2R cells at 4 h after 4 Gy IR or non-IR. **C**, **D** Western blot assay of SLC7A11, GPX4 and SOCS2 proteins in SK-Hep-1, SK-Hep-1R, HepG2 and HepG2R cells with or without lvSOCS2 transfection at 4 h after 4 Gy IR or non-IR. Representative images (**F**) and quantification (**E**) of the relative fluorescence intensity of liperfluo in SK-Hep-1 and HepG2 cells transfected with lvSOCS2 at 4 h after 4 Gy IR or non-IR. Nuclei were stained with Hoechst (x40). Scale bars, 10 μm. *P < 0.05, **P < 0.01 and ***P < 0.001 between indicated groups.

Since both ferroptosis and SOCS2 contributed to radiosensitization of HCC, and Fig. 2J implied a promotion of ferroptosis by SOCS2, we tried to probe the relationship between SOCS2 and ferroptosis deeply. It was determined that SOCS2 increased while GPX4 decreased within 24 h post-IR compared to unirradiated cells, with the most pronounced change at 4 h (Fig. 4A). Next, we measured the protein expressions in unirradiated or 4 h post-IR cells, and found that SOCS2 attenuated with the increase of radioresistance, in contrast to GPX4 and SLC7A11 (Fig. 4B; Fig. S4A). Moreover, the increment of SOCS2 reduced GPX4 and SLC7A11 in both non-irradiated and irradiated cells (Fig. 4C, D; Fig. S4B, C).

Above outcomes suggested that SOCS2 promoted the occurrence of ferroptosis at protein level, so we proceeded to explore the relationship between SOCS2 and characteristic phenotypes of ferroptosis: intracellular Fe^2+^ content and lipid peroxides. Figure 4E, F revealed that increasing SOCS2 advanced the accumulation of cytoplasmic lipid peroxides in HCC cells, more noticeable post-IR. However, Fer-1 could inhibit the onset of SOCS2-induced ferroptosis (Fig. S4D, E). Besides, the Fe^2+^ content was remarkably augmented in HCC cells with overexpressed SOCS2, especially upon IR (Fig. S4F). Broadly, SOCS2 suppressed the GPX4 and SLC7A11 expressions and induced the accumulation of lipid peroxides and Fe^2+^ to contribute to ferroptosis.

### SOCS2 degraded SLC7A11 by ubiquitination

**Fig. 5:**
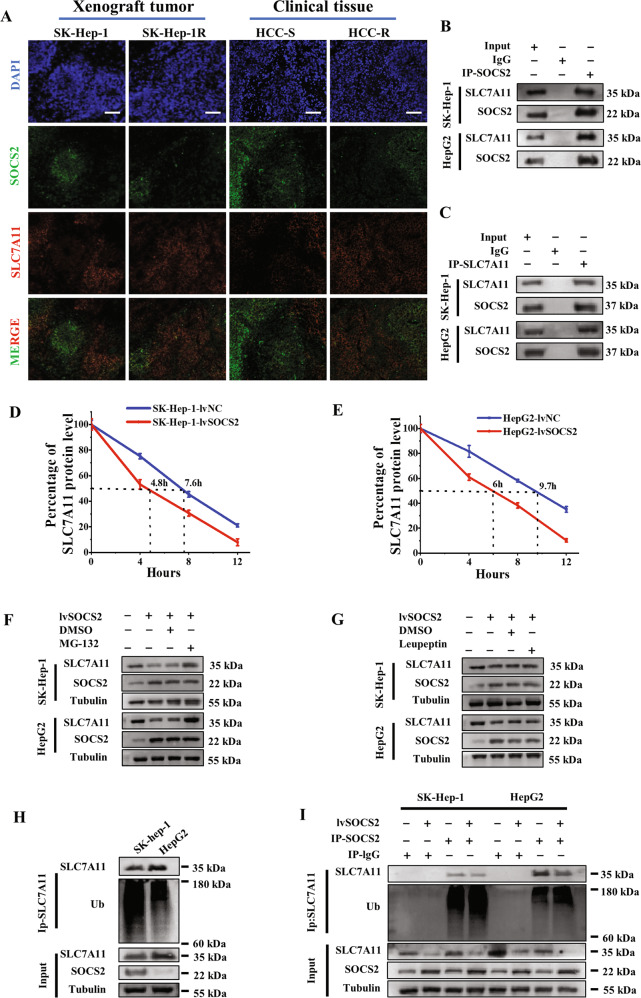
SOCS2 interacted with SLC7A11 and decreased its level by ubiquitin-proteasome pathway. **A** Representative immunofluorescence images of SOCS2 and SLC7A11 proteins in the nonirradiated xenograft tumors (SK-Hep-1 and SK-Hep-1R) and HCC clinical tissues. Nuclei are stained with DAPI (×10). Scale bars, 100 μm. **B**, **C** Co-immunoprecipitation and Western blot assay of SOCS2 and SLC7A11 proteins in the whole cell lysates of SK-Hep-1 and HepG2 cells at 4 h after 4 Gy IR. **D**, **E** Point-fold line chart of SLC7A11 protein degradation according to Western blot assay (Fig. S5E). **F**, **G** Western blot analysis of SLC7A11, SOCS2 and tubulin proteins in SK-Hep-1 and HepG2 cells at 4 h after 4 Gy IR. MG-132 (10 μM) or leupeptin (50 μM) were added before IR. **H** Anti-Ub immunoblotting assay of SLC7A11 polyubiquitination in SK-Hep-1 and Hep2 cells at 4 h after 4 Gy IR. **I** Anti-Ub immunoblotting assay of SLC7A11 polyubiquitination in SK-Hep-1 and HepG2 cells transfected with lvSOCS2 at 4 h after 4 Gy IR. *P < 0.05, **P < 0.01 and ***P < 0.001 between indicated groups.

To investigate how SOCS2 mediates ferroptosis and thus regulates HCC radiosensitivity, we analyzed the co-expressed protein network of SOCS2 using HCCDB database. Figure S5A illustrated that 19 proteins, including the solute carrier protein family members of SLC25A15 and SLC1A1, were co-expressed with SOCS2. Although SLC7A11 was not displayed in this network, as another member of the solute carrier protein family, we hypothesized that SLC7A11 might also associate with SOCS2. A predictive analysis of the correlation between SOCS2 and SLC7A11 in HCC and paraneoplastic tissues using GEPIA database showed that SOCS2 was negatively correlated with SLC7A11 with a correlation coefficient of 0.32 (Fig. S5B). Further immunofluorescence staining of SOCS2 and SLC7A11 proteins in xenografts (with or without IR) and clinical tissues also illustrated that the fluorescence density of SOCS2 was opposite to that of SLC7A11, with negative correlation coefficients of 0.4843, 0.622, 0.5439, 0.6728, 0.5221 and 0.6711, respectively (Fig. 5A; Fig. S5C, D).

To verify whether SLC7A11 and SOCS2 could interact each other directly, we extracted whole cell lysates and then co-isolated SOCS2 protein and its interacting proteins by immunoprecipitation. As expected, SLC7A11 was detected in SOCS2 interactive proteins. Similarly, SOCS2 was also identified in SLC7A11-interacting proteins (Fig. 5B, C). To understand the functional consequences of this interaction, we wondered whether SOCS2 could negatively regulate SLC7A11 expression or induce its degradation. Indeed, after overexpression of SOCS2, the steady-state level of SLC7A11 was markedly reduced (Fig. S5E) and the half-life of SLC7A11 was considerably shortened (Fig. 5D, E). However, the transcript level of SLC7A11 did not change dramatically in HCC cells (Fig. S5F), implicating that SOCS2 promoted SLC7A11 degradation via the protein aspect instead of transcriptional aspect. Next, to investigate the mechanism of SLC7A11 degradation thoroughly, we added a proteasome inhibitor (MG-132) and lysosome inhibitor (leupeptin) to HCC cells. Although high-expressed SOCS2 could reduce the protein level of SLC7A11, this downregulation was reversed by the treatment of MG-132 whereas it failed to be reversed by leupeptin (Fig. 5F, G; Fig. S5G, H), suggesting that descending SLC7A11 induced by SOCS2 was dependent on proteasomal pathway rather than lysosomal pathway. Since SOCS2 served as an E3 ubiquitin ligase, we speculated that SLC7A11 might be specifically recognized by SOCS2 and underwent ubiquitinated degradation. Western blotting assay demonstrated that the level of ubiquitinated SLC7A11 in SK-Hep-1 cells was distinctly higher than that in HepG2 cells (Fig. 5H; Fig. S5I). What’s more, a prominent depletion of SLC7A11 and a more pronounced level of ubiquitinated SLC7A11 were observed in HCC cells with high-expressed SOCS2 (Fig. 5I; Fig. S5J).

### SH2-domain of SOCS2 interacted with N-terminal domain of SLC7A11 to promote radiosensitization

**Fig. 6:**
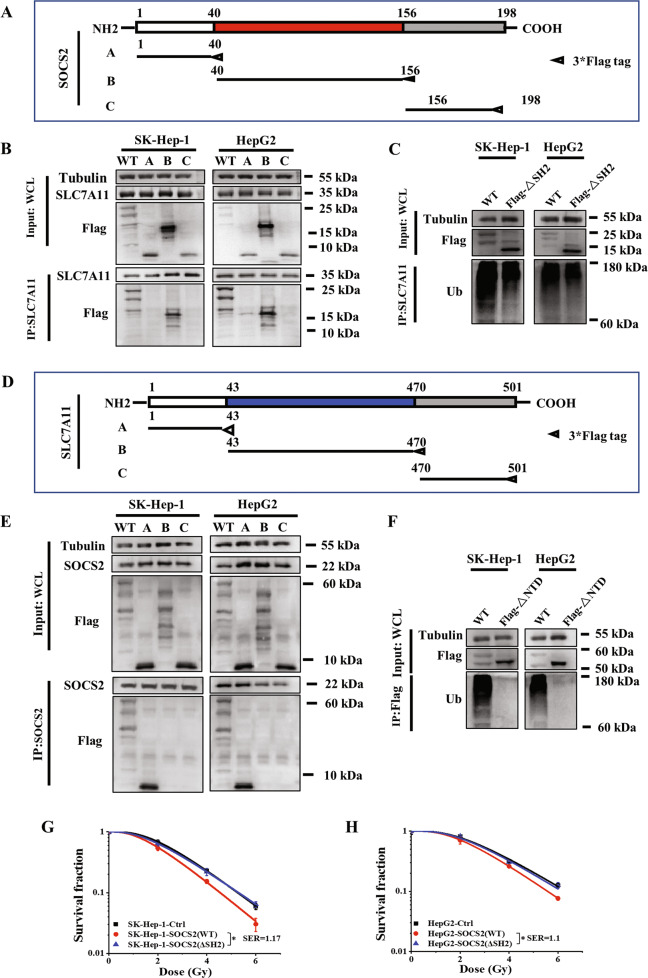
SOCS2-SH2 domain interacted with N-terminal domain of SLC7A11 to reduce radioresistance. **A** Schematic representation of three flag-fused SOCS2 constructs containing amino acids 1–40, 40–156 and 156–198. **B** Co-immunoprecipitation assay of SLC7A11 and different SOCS2 domains in the whole cell lysates of irradiated SK-Hep-1 and HepG2 cells. **C** Ubiquitination of SLC7A11 in irradiated SK-Hep-1 and HepG2 cells with or without ΔSH2 manipulation (ΔSH2, SH2 domain truncation mutant of SOCS2). **D** Schematic representation of three flag-fused SLC7A11 constructs containing amino acids 1–43, 43–470 and 470–501. **E** Co-immunoprecipitation assay of SOCS2 and different SLC7A11 domains in the whole cell lysates of irradiated SK-Hep-1 and HepG2 cells. **F** SLC7A11 polyubiquitination was detected by anti-Ub immunoblotting in 4 Gy irradiated SK-Hep-1 and HepG2 cells with or without ΔNTD manipulation (ΔNTD, NTD domain truncation mutant of SOCS2). **G**, **H** Dose responses of survival fractions of SK-Hep-1 and HepG2 cells transfected with SOCS2-ΔSH2 or SOCS2-WT plasmid. *P < 0.05, **P < 0.01 and ***P < 0.001 between indicated groups.

To clarify the interaction region between SOCS2 and SLC7A11, we applied two protein structure analysis websites (NCBI: http://www.ncbi.nlm.nih.gov/ and UniProt: https://www.uniprot.org/) to classify SOCS2 into three structural domains: NTD, SH2-structural and CTD, corresponding to the amino acid sequences at 1–40, 40–156 and 156–198, respectively. We then constructed three truncation plasmids (plasmid A, B, C) based on above structural domain sequences and a wild-type (WT) plasmid containing full SOCS2 sequence with 3×Flag tags at C-termini and transfected these plasmids into HCC cells (Fig. 6A). Immunoprecipitation and immunoblotting assay illustrated that only the channels of WT and B (SH2 truncation) exhibited SLC7A11 bands, indicating that SLC7A11 interacted with the SH2-structural domain of SOCS2 (Fig. 6A). Afterwards, to investigate the impact of SOCS2-SH2 domain on the level of ubiquitination, we generated a SOCS2-ΔSH2 plasmid by deleting the SH2-structural domain from SOCS2 full sequence and found that SOCS2-ΔSH2 mutant could not increase ubiquitinated SLC7A11, whereas SOCS2-WT plasmid significantly increased the ubiquitination level of SLC7A11 (Fig. 6C). Therefore, SOCS2 induced SLC7A11 ubiquitination through SH2-structural domain of SOCS2.

Moreover, to identify the structural domain of SLC7A11 interacting with SOCS2, we also established three truncation plasmids (plasmid A, B, C corresponding to the amino acid sequences at 1–43, 43–470, 470–501, respectively) based on the structural domains of SLC7A11 (Fig. 6D) and found that SOCS2 interacted with the NTD of SLC7A11 (Fig. 6E). Consistent with the construction of SOCS2-ΔSH2 plasmid, we also constructed SLC7A11-ΔNTD mutant plasmids without N-terminal. Surprisingly, the level of ubiquitination of Flag-SLC7A11 was undetectable while a high level of ubiquitinated Flag-SLC7A11 still presented in WT group (Fig. 6F), indicating that SLC7A11-NTD interacted with SOCS2 and consequently mediated its own ubiquitination. Furthermore, the survival fraction of SOCS2-ΔSH2 group had no significant difference in comparison with its negative control but significantly higher than that of SOCS2-WT group (Fig. 6G, H), implying that SOCS2 exerts a radiosensitization effect in HCC cells due to its SH2-structural domain.

### SOCS2 promoted K48-related polyubiquitination of SLC7A11 and bound to elongin B/C

**Fig. 7:**
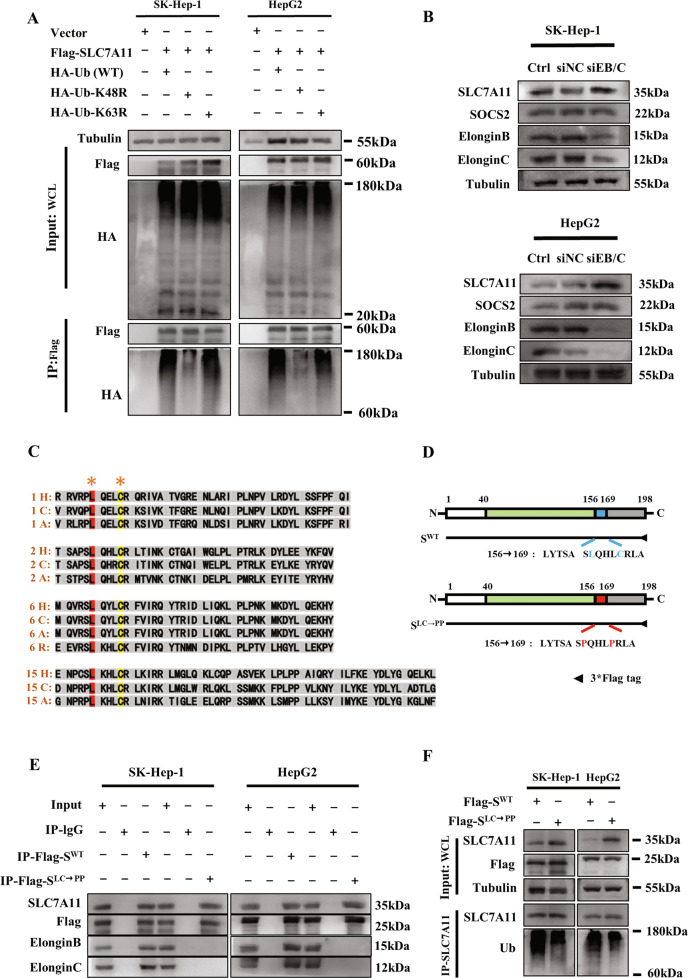
SOCS2 mediated K48-linked polyubiquitination chains onto SLC7A11 via an elongin B/C ubiquitin-complex. **A** Immunoprecipitation and immunoblotting assay were selected to detect the type of polyubiquitination of Flag-SLC7A11 in SK-Hep-1 and HepG2 cells transfected with wild-type or K48/K63 mutant Ub after IR. **B** Western blot assay of SLC7A11, elongin B and elongin C proteins in 4 Gy irradiated SK-Hep-1 and HepG2 cells transfected with siRNA targeting *elongin B* or *elongin C* (*siEB/C*). **C** Amino acid sequences of SOCS2-BOX regions of SOCS1 (1), SOCS2 (2), SOCS6 (6) and SAB15 (15) proteins from different species. H refers to Human; C refers to Chicken; A refers to African clawed frog; R refers to Red flour beetle. **D** Schematic representation of two flag-fused SOCS2 plasmids with wild-type (S^WT^) or L162C166/P162P166 (S^LC→PP^) mutant. **E** Co-immunoprecipitation assay of S^WT^, S^LC→PP^, elongin B and elongin C in the whole cell lysates of irradiated SK-Hep-1 and HepG2 cells. **F** SLC7A11 polyubiquitination was detected by anti-Ub immunoblotting assay in 4 Gy irradiated SK-Hep-1 and HepG2 cells transfected with S^WT^ or S^LC→PP^ plasmid.

After clarifying that SOCS2-SH2 could mediate SLC7A11 ubiquitination by recognizing SLC7A11-NTD, we sought what type of polyubiquitination occurred in SLC7A11. So far, K48- and K63-linked chains have been reported as the most abundant and functionally well-characterized polyubiquitin chains [33, 34]. To determine which of them was presented in polyubiquitinated SLC7A11, we designed ubiquitin mutants at 48/63 lysine of arginine (K48R/K63R) to specifically prevent the formation of K48- or K63-linked chain. It was found that when the amount of precipitated Flag-SLC7A11 was consistent, HA-Ub-K48R inhibited the accumulation of polyubiquitinated SLC7A11 after IR, whereas HA-Ub-K63R had no such effect (Fig. 7A), implying that the SLC7A11 polyubiquitination was mainly produced in the form of K48-linked ubiquitin chain instead of K63-linked. Notably, the K48R mutation reduced the level of SLC7A11 polyubiquitination but not completely, hinting that other linkage types might also contribute to this polyubiquitination

As mentioned previously, SOCS2 and SOCS6 generally bound to elongin B/C by SOCS-BOX region to co-mediate ubiquitination, we then investigated whether the SOCS2-mediated SLC7A11 ubiquitination in HCC cells also required the involvement of elongin B/C. Figures 7B and S6A revealed that knockdown of elongin B/C did not impact SOCS2 but elevated SLC7A11 in HCC cells, suggesting that elongin B/C complex was required for SOCS2-mediated ubiquitination degradation of SLC7A11.

We next explored which amino acid site of SOCS2 interacted with elongin B/C complex. By comparing the amino acid sequences of SOCS-BOX region in SOCS2 with those in SOCS1, SOCS6 and ASB15 (another protein containing SOCS-BOX region) using ClustalW tool, we found only leucine (Leu, L) and cysteine (Cys, C) consistently existed among different proteins (Fig. 7C). Besides, the predicted secondary structures of these amino acid sequences in SOCS2-BOX region showed that Leu and Cys were almost engaged in α-helix process (Fig. S6C), signifying that these two amino acids were highly conserved in primary sequence and functionally similar in secondary structure. Consequently, we hypothesized that Leu and Cys might be the target sites of the interaction of SOCS2 with elongin B/C. To verify this conjecture, we constructed a SOCS2 mutant plasmid (SOCS2^LC→PP^) where Leu 162 (L162) and Cys 166 (C166) of SOCS2 were both mutated to proline (Pro, P), as well as a SOCS2^WT^ plasmid as control (Fig. 7D). Figures 7E and S6B showed that Flag-SOCS2^WT^ could interact with SLC7A11, elongin B and elongin C; while SOCS2^LC→PP^ only interacted with SLC7A11 but failed to precipitate elongin B and elongin C. Furthermore, SOCS2^LC→PP^ also accounted for the reduction of ubiquitination SLC7A11 (Fig. 7F), implying that elongin B/C interacted with SOCS2 at L162 and C166 sites to forge the SOCS2/elongin B/C complex and thus co-facilitated the ubiquitinated degradation of SLC7A11.

## Discussion

Previous studies have confirmed that radiotherapy facilitated the occurrence of ferroptosis [30, 31], whereas this research revealed that ferroptosis could conversely enhance the radiosensitivity of HCC tissues or cells. Meanwhile, SOCS2 was identified to predict and increase the radiosensitivity of HCC. In both HCC tissues and transplanted tumors, we found a robust negative correlation between SOCS2 and SLC7A11 and subsequently confirmed that SOCS2 served as a specific E3 ubiquitin ligase for SLC7A11, promoted ferroptosis by mediating the SLC7A11 degradation, and ultimately led to radiosensitization.

For SOCS2, its SH2-domain and SOCS-box were most closely associated with the ubiquitination of SLC7A11 [35]. The SH2 domain was a crucial structural-domain for the recognition of SLC7A11, while the BOX region bound to elongin B/C complex to form an E3 ubiquitin ligase complex to recruit ubiquitin molecules. Herein, we hypothesized that SOCS2 could serve as a bridge between SLC7A11 and ubiquitin, utilizing its SH2 region to recognize certain phosphorylated tyrosine residues [36, 37] at SLC7A11-NTD, recruiting ubiquitin through BOX region, thereby transferring ubiquitin to SLC7A11.

Furthermore, most ubiquitination usually occurs on the lysine of substrate protein [34, 38]. Using the ClustalW tool, our sequence analysis of SLC7A11 protein from different species revealed that seven conserved lysine sites could potentially to be ubiquitinated (Supplementary document S1), pending for additional investigation. Regarding to SOCS2-BOX domain, it was confirmed to constitute an E3 ligase complex (SOCS2/ elongin B/C) with the elongin B/C compound through L162 and C166 amino acid sites, which was well preserved in the amino acid sequences of SOCS1, SOCS6 and SAB15 to mediate ubiquitination.

**Fig. 8:**
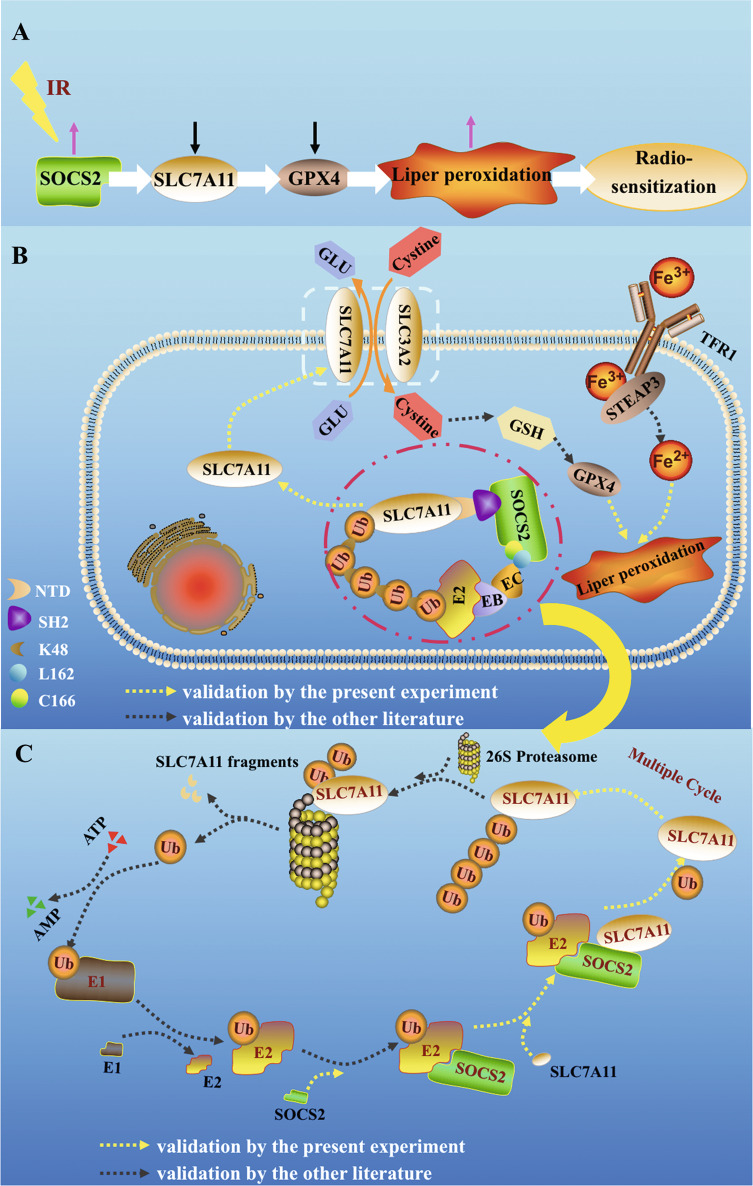
Incremental SOCS2 promoted ubiquitination degradation of SLC7A11 and induced ferroptosis and radiosensitization of HCC. **A** The pathway of SOCS2 sensitization. After IR, a rapid increase of SOCS2 led to a decrease of SLC7A11 and advanced ferroptosis and radiosensitization. **B** Diagram of the specific mechanism of SOCS2-induced ferroptosis. SOCS2-SH2 recognized SLC7A11-NTD, bound ubiquitin molecules which was linked to E2 ubiquitin-binding enzymes with the joint involvement of elongin B (EB) and elongin C (EC), and facilitated the transfer of ubiquitin molecules to the substrate protein SLC7A11 to promote SLC7A11 degradation. This degradation subsequently caused a reduction in cystine intake as well as a reduction in GSH and GPX4 and ultimately advanced ferroptosis. **C** The processes of SLC7A11 protein ubiquitination. In an ATP-dependent reaction, Ub was attached to the ubiquitin-activating enzyme E1 and activated, then delivered to the ubiquitin-binding enzyme E2. Next, the E3 ligase SOCS2 acted as a bridge, identifying E2-Ub and the substrate SLC7A11 to facilitate the association of Ub with SLC7A11. After several repetitions of the above process, a K48-ubiquitin chain was formed and attached to SLC7A11. Finally, the K48-ubiquitin chain was identified by the 26 S proteasome, leading to the degradation of SLC7A11 to fragments. Yellow arrows indicate the experimental results argued in this paper while black arrows represent results reported in other literature.

Based on present results, we would outline the processes of SOCS2-mediated HCC radiosensitization by promoting ferroptosis. IR increases SOCS2 expression, followed by the binding of SOCS2-SH2 to SLC7A11-NTD and the binding of L162 and C166 in SOCS2 to elongin B/C, recruitment of ubiquitin, and induction of SLC7A11 K48-chain-linked-polyubiquitination (Fig. 8A, B). Eventually, SLC7A11 with ubiquitin chains is recognized by 26 S proteasome and disassembled into protein fragments (Fig. 8C) for degradation [39], which ultimately contributes to the onset of HCC ferroptosis and radiosensitization.

This study focused on the mechanism of which SOCS2 facilitated the degradation of ubiquitinated SLC7A11 and thus increased the formation of ferroptosis, although the intracellular Fe^2+^ level was also observed to be increased in SOCS2 over-expressed cells, which has not been reported yet. It was reported that STEAP3 was significantly downregulated in HCC tissues and its lower level represented poor prognosis [40]. Mechanically, STEAP3 has been demonstrated as a major reductant for the reduction of Fe^3+^ to Fe^2+^ [41] and is a target gene of P53[42]. Since the SOCS family could promote P53 expression [43–45], we speculate that SOCS2 may also induce the formation of Fe^2+^ by enhancing the expression of STEAP3, thereby promoting ferroptosis and could further investigated it in future. In conclusion, our findings suggest that SOCS2 may be capable of a potential biomarker for predicting the prognosis of radiotherapy as well as a potential new therapeutic target for enhancing radiosensitivity of HCC.

## Supplementary information

Supplementary figures 1 to 6

Supplementary table 1

Supplementary table 2

Supplementary table 3

Supplementary table 4

Supplementary table 5

Supplementary document

Reproducibility checklist

## Data Availability

The data supporting the present study are available from the corresponding author upon reasonable request.
