# Supplementary material for: SOCS2-enhanced ubiquitination of SLC7A11 promotes ferroptosis and radiosensitization in hepatocellular carcinoma
Source: Cell Death Differ. 2022 Aug 22;30(1):137–51. doi: 10.1038/s41418-022-01051-7 (PMC9883449; doi:10.1038/s41418-022-01051-7)
Supplement: Supplementary file 1 — Supplementary figures 1 to 6 [file 41418_2022_1051_MOESM1_ESM.docx]

**SOCS2-enhanced ubiquitination of SLC7A11 promotes ferroptosis and radiosensitization in hepatocellular carcinoma**

Qianping Chen^1#^, Wang Zheng^1#^, Jian Guan^2#^, Hongxia Liu^1^, Yao Dan^1^, Lin Zhu^3^, Yimeng Song^1^, Yuchuan Zhou^1^, Xinrui Zhao^1^, Yuhong Zhang^1^, Yang Bai^1^, Yan Pan^1^*, Jianghong Zhang^1^*, Chunlin Shao^1^*

^1^Institute of Radiation Medicine, Shanghai Medical College, Fudan University, Shanghai 200032, China;

^2^Department of Radiation Oncology, Nanfang Hospital of Southern Medical University, Guangzhou, Guangdong 510515, China

^3^Department of Radiation Oncology, Shanghai Cancer Center, Shanghai Medical College, Fudan University, Shanghai 200032, China

**# These authors contributed equally to this work**

***Authors for correspondence:**

Prof. Chunlin Shao, E-mail: [clshao@shmu.edu.cn](mailto:clshao@shmu.edu.cn).

Dr. Jianghong Zhang, E-mail: [zjh551268@fudan.edu.cn](mailto:zjh551268@fudan.edu.cn).

Dr. Yan Pan, E-mail: [Swallowpan@fudan.edu.cn．](mailto:Swallowpan@fudan.edu.cn．)

**Supplementary Figures S1-S6**

**
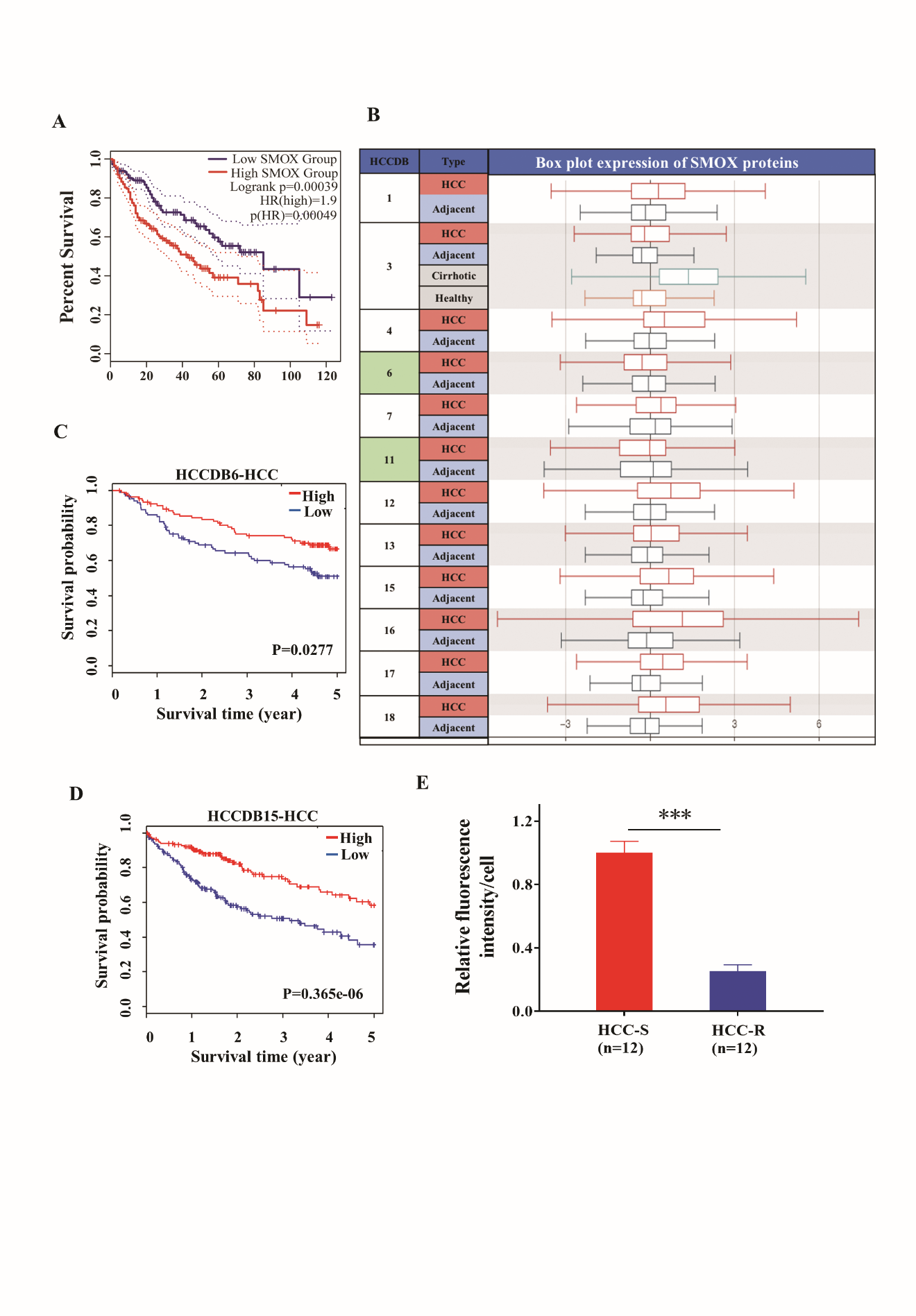
**

**Fig. S1**. High expression of *SOCS2* and low expression of *SMOX* both responded to better prognosis in HCC, but *SMOX* was partially overexpressed in HCC compared to adjacent tissues. **(A)** Kaplan-Meier curve of HCC survival analysis based on the expression status of *SMOX* gene according to TCGA and GEPIA dataset. **(B)**. Box scatter diagram of the expression of *SMOX* in tumor tissues and their adjacent normal tissues in HCCDB. The central mark was the median and the edges of the box were the 25th and 75th percentiles. **(C, D)** Kaplan-Meier curve of HCC survival analysis based on the expression status of *SOCS2* gene according to HCCDB6 and HCCDB15. **(E)** Quantification of the relative fluorescence intensity of SOCS2 protein in the tumor tissues of radioresistant HCC patients (HCC-R) (n=12) and radiosensitive HCC patients (HCC-S) (n=12, see Fig 1I). * *P* < 0.05, ** *P* < 0.01 and *** *P* < 0.001 between indicated groups.

**
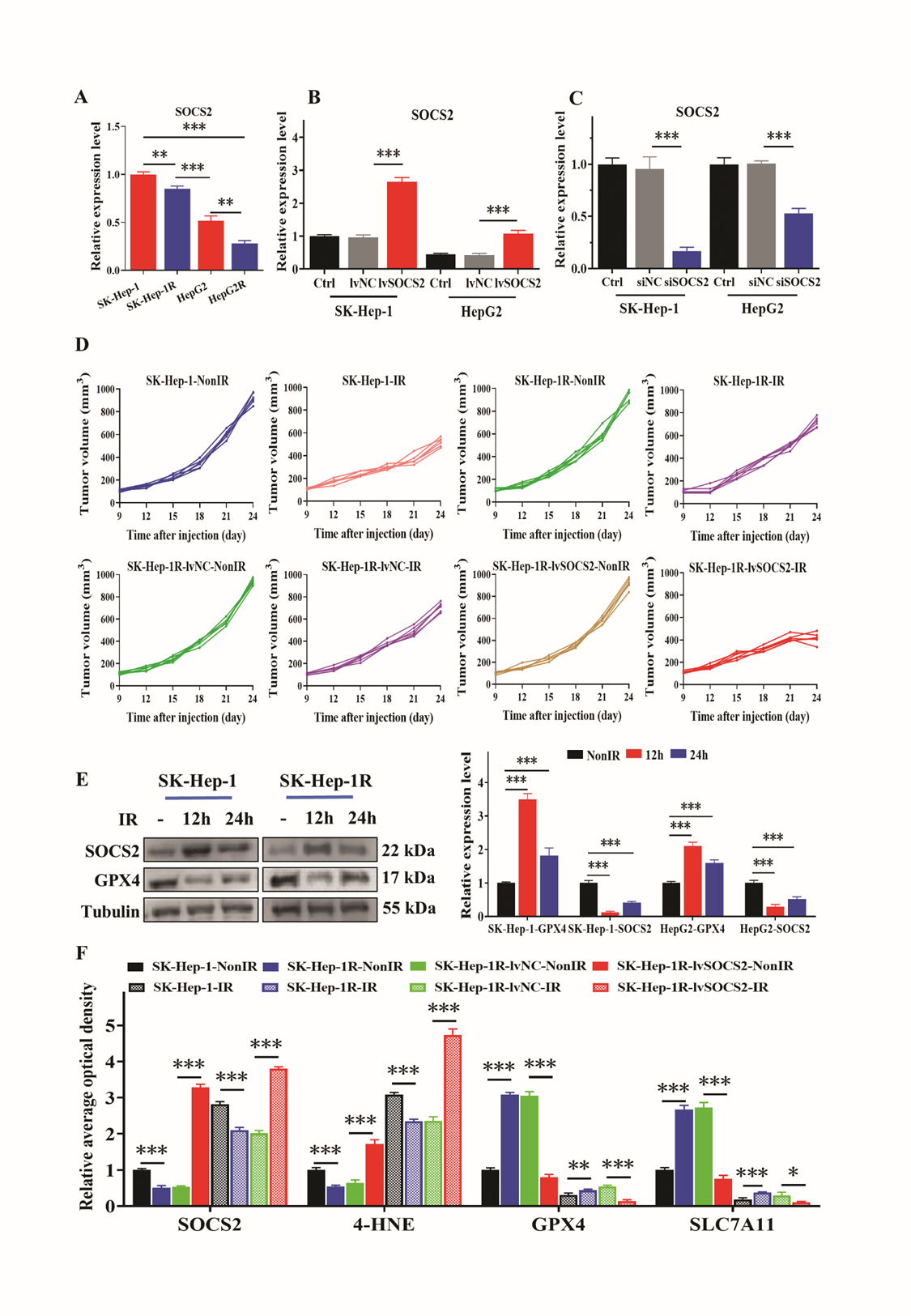
Fig. S2**. High expression of SOCS2 could act as a radiosensitizer. **(A)** Relative expression level of SOCS2 protein in SK-Hep-1, SK-Hep-1R, HepG2 and HepG2R cells (see Fig. 2A). **(B)** Relative expression level of SOCS2 protein in SK-Hep-1 and HepG2 cells transfected with lvSOCS2 or their negative control (lvNC) (see Fig. 2B). **(C)** Relative expression level of SOCS2 protein in SK-Hep-1 and HepG2 cells transfected with si*SOCS2* or its negative control (siNC) (see Fig. 2C). * *P* < 0.05, ** *P* < 0.01 and *** *P* < 0.001 between indicated groups. **(D)** In each group (SK-Hep-1-NonIR, SK-Hep-1-IR, SK-Hep-1R-NonIR, SK-Hep-1R-IR, SK-Hep-1R-lvNC-NonIR, SK-Hep-1R-lvNC-IR, SK-Hep-1R-lvSOCS2-NonIR, and SK-Hep-1R-lvSOCS2-IR), the volume of transplanted tumor from each nude mouse was examined every 3 days until 24 days after subcutaneous injection. (**E**) Western Blot assay of GPX4, SOCS2 and Tubulin proteins and their relative levels in SK-Hep-1 and SK-Hep-1R transplanted tumors at 12 and 24 h after IR or non-IR. (**F**) Relative expressions of SOCS2, 4-HNE, GPX4 and SLC7A11 protein (see Fig. 2J) were quantified by Image J in the manner of average optical density (AOD) (x 40) in the aforementioned xenograft tumors (n=6 in each group). * *P* < 0.05, ** *P* < 0.01 and *** *P* < 0.001 between indicated groups.


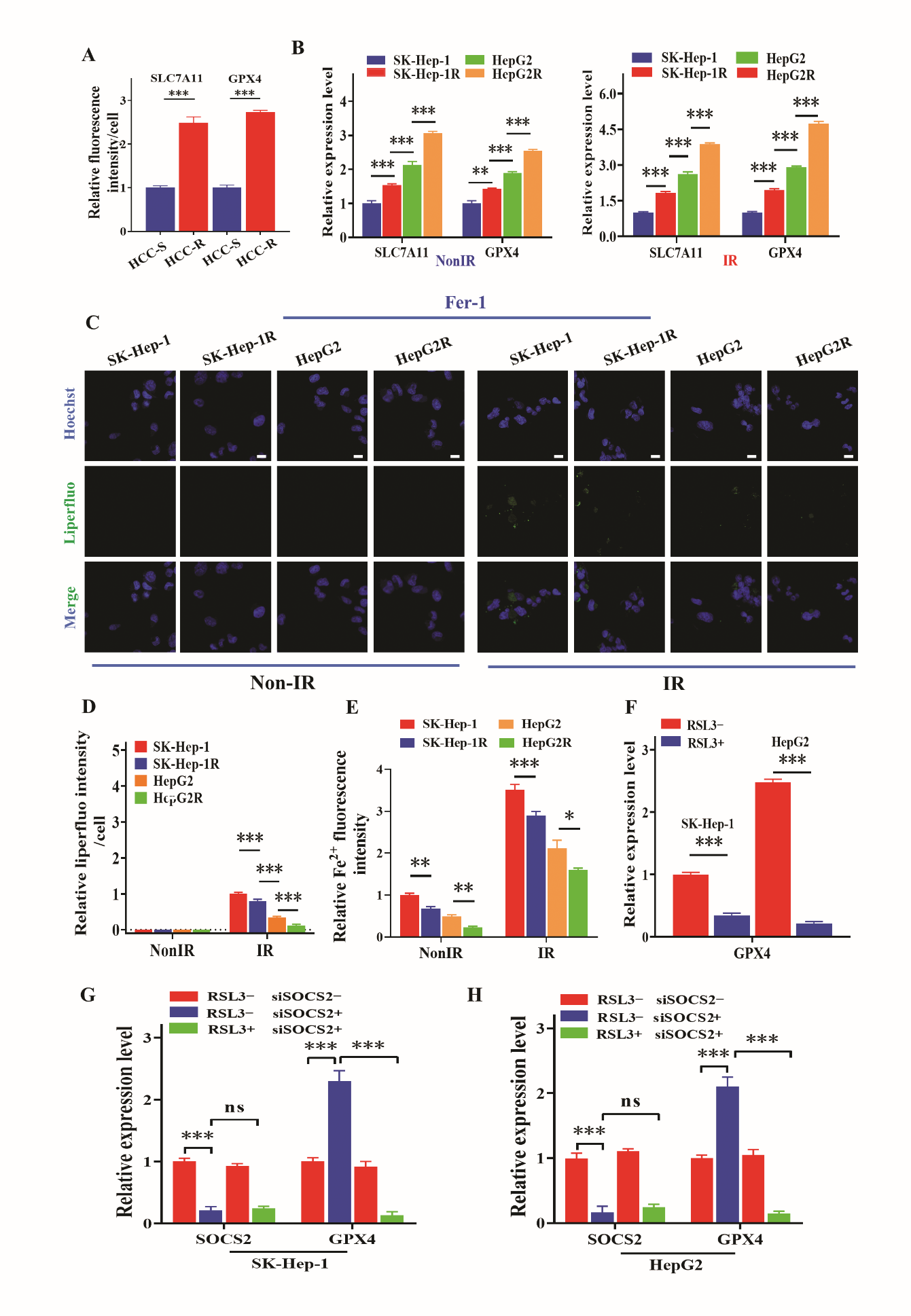
**Fig. S3**. Accelerated ferroptosis caused HCC cells or tissues to be more sensitive to IR. **(A)** Quantification of fluorescence intensity of SLC7A11 and GPX4 proteins assessed by immunofluorescence assay in HCC clinical tissues from 12 radioresistant (HCC-R) and 12 radiosensitive (HCC-S) patients (see Fig. 3A). **(B)** Relative expression levels of SLC7A11 and GPX4 proteins in SK-Hep-1, SK-Hep-1R, HepG2 and HepG2R cells at 4 h after IR (4Gy) or NonIR (see Fig. 3B). (**C, D**) Representative images (C) and quantification (D) of the relative fluorescence intensity of liperfluo in SK-Hep-1, SK-Hep-1R, HepG2 and HepG2R cells at 4 h after 4 Gy IR or non-IR with the addition of Fer-1. Nuclei are stained with Hoechst (x40). Scale bars, 10 μm. **(E)** Relative level of intracellular Fe^2+^ in SK-Hep-1, SK-Hep-1R, HepG2 and HepG2R cells at 4 h after 4 Gy IR or non-IR (see Fig. 2J). **(F)** Relative expression level of GPX4 protein in SK-Hep-1 and HepG2 cells with or without RSL3 treatment (see Fig. 3E). **(G, H)** Relative expression levels of GPX4 and SOCS2 protein in SK-Hep-1 (see Fig. 3F) and HepG2 cells (see Fig. 3G) treated with RSL3, si*SOCS2* or siNC. * *P* < 0.05, ** *P* < 0.01 and *** *P* < 0.001 between indicated groups**.**

**
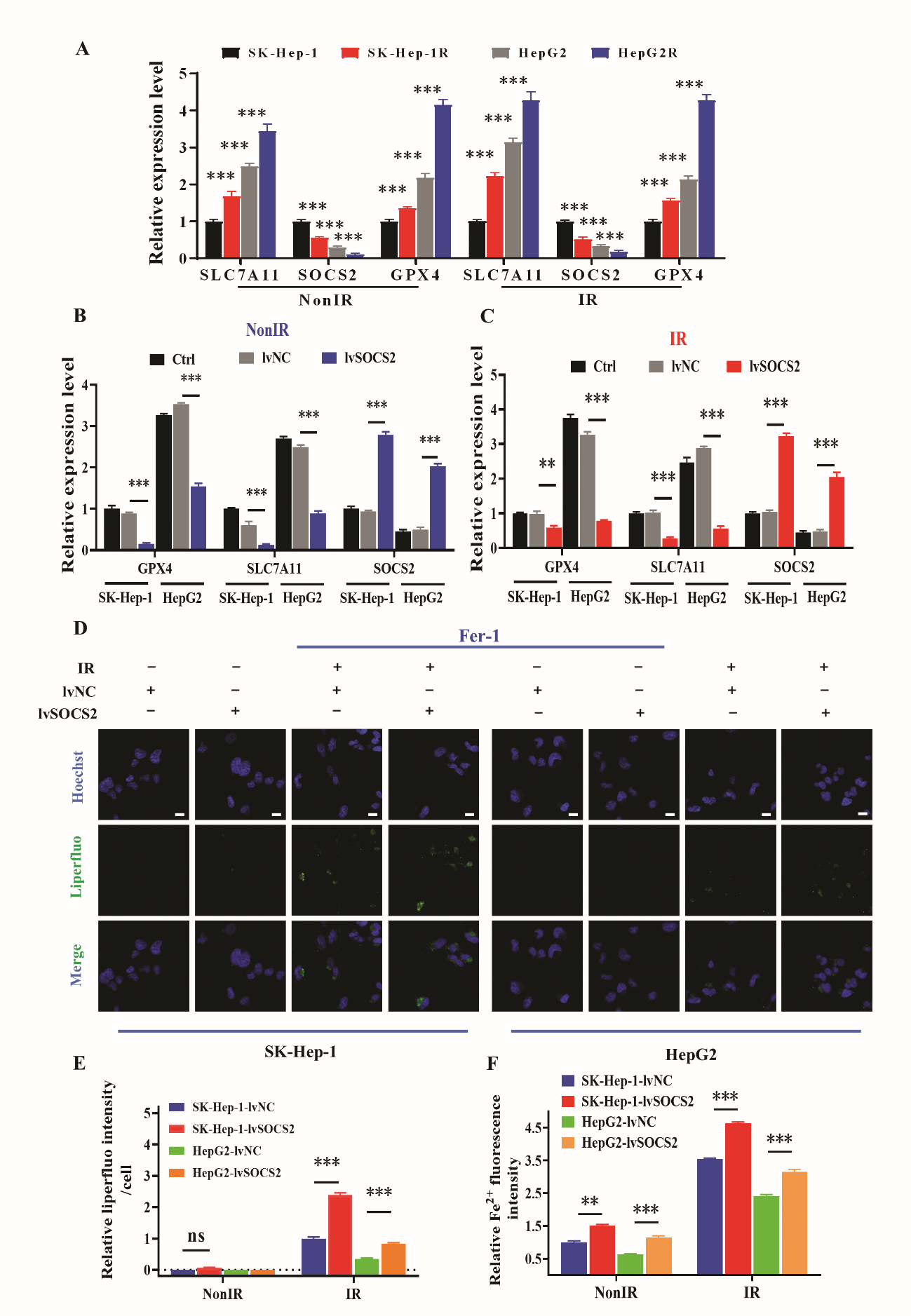
**

**Fig. S4**. Elevation of SOCS2 promoted the onset of ferroptosis. **(A)** Relative expression levels of SLC7A11, GPX4 and SOCS2 proteins in SK-Hep-1, SK-Hep-1R, HepG2 and HepG2R cells at 4 h after 4Gy IR or non-IR (see Fig. 4B). **(B, C)** Relative expression levels of SLC7A11, GPX4 and SOCS2 proteins in SK-Hep-1 and HepG2 cells transfected with lvSOCS2 or its negative vector (lvNC) at 4 h after 4 Gy IR or non-IR (see Fig. 4C, D). (**D, E**) Representative images (E) and quantification (D) of the relative fluorescence intensity of liperfluo in SK-Hep-1 and HepG2 cells transfected with lvSOCS2 at 4 h after 4Gy IR or non-IR with the treatment of Fer-1. Nuclei are stained with Hoechst (x40). Scale bars, 10 μm. **(F)** Relative level of intracellular Fe^2+^ in SK-Hep-1 and HepG2 cells transfected with lvSOCS2 at 4 h after 4 Gy IR or non-IR. * *P* < 0.05, ** *P* < 0.01 and *** *P* < 0.001 between indicated groups.


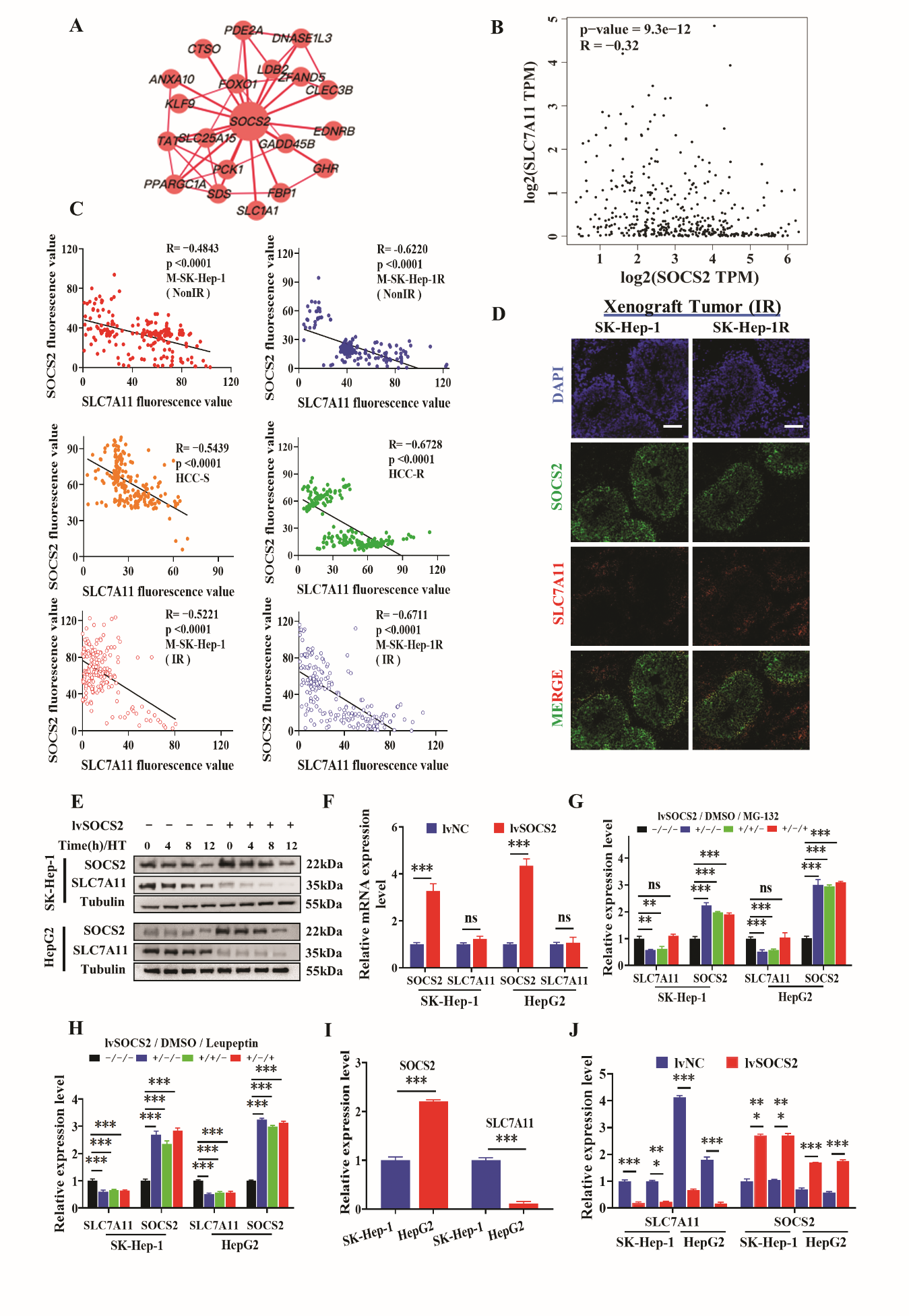


**Fig. S5.** SOCS2 was negatively linked to the expression of SLC7A11. **(A)** Meta-analysis of SOCS2 co-expression network using HCCDB dataset. **(B)** Pearson’s correlation of SOCS2 and SLC7A11 in HCC tissues and paracancerous tissues from GEPIA and TCGA dataset. **(C)** Pearson analysis and regression analysis of the correlation between SOCS2 and SLC7A11 in xenograft tumors (SK-Hep-1 and SK-hep-1R) with or without IR and HCC clinical tissues. M refers to xenograft tumors from nude mice. n=6 (M-SK-Hep-1); n=6 (M-SK-Hep-1R); n=12 (HCC-S); n=12 (HCC-R). (**D**) Representative immunofluorescence images of SOCS2 and SLC7A11 proteins in the xenograft tumors (SK-Hep-1 and SK-Hep-1R) at 12 h post-IR. Nuclei are stained with DAPI (x10). Scale bars, 100 μm. **(E)** At different time points after 4 Gy IR, the expressions of SLC7A11 and SOCS2 protein in SK-Hep-1 and HepG2 cells with or without lvSOCS2 transfection after harringtonine (HT) treatment. **(F)** Relative mRNA levels of *SLC7A11* and *SOCS2* in the irradiated SK-Hep-1 and HepG2 cells with or without lvSOCS2 transfection. **(G, H)** Relative expression levels of SLC7A11 and SOCS2 proteins in SK-Hep-1 and HepG2 cells treated with MG132 (10 μM, see Fig. 5F) or Leupeptin (50 μM, see Fig. 5G) at 4 h after 4 Gy IR. **(I)** Relative expression levels of SLC7A11 and SOCS2 proteins in SK-Hep-1 and HepG2 cells at 4 h after 4 Gy IR (see Fig. 5H). **(J)** Relative expression levels of SLC7A11 and SOCS2 proteins in SK-Hep-1 and HepG2 cells with or without lvSOCS2 transfection at 4 h after 4Gy IR (see Fig. 5I). * *P* < 0.05, ** *P* < 0.01 and *** *P* < 0.001 between indicated groups.

**
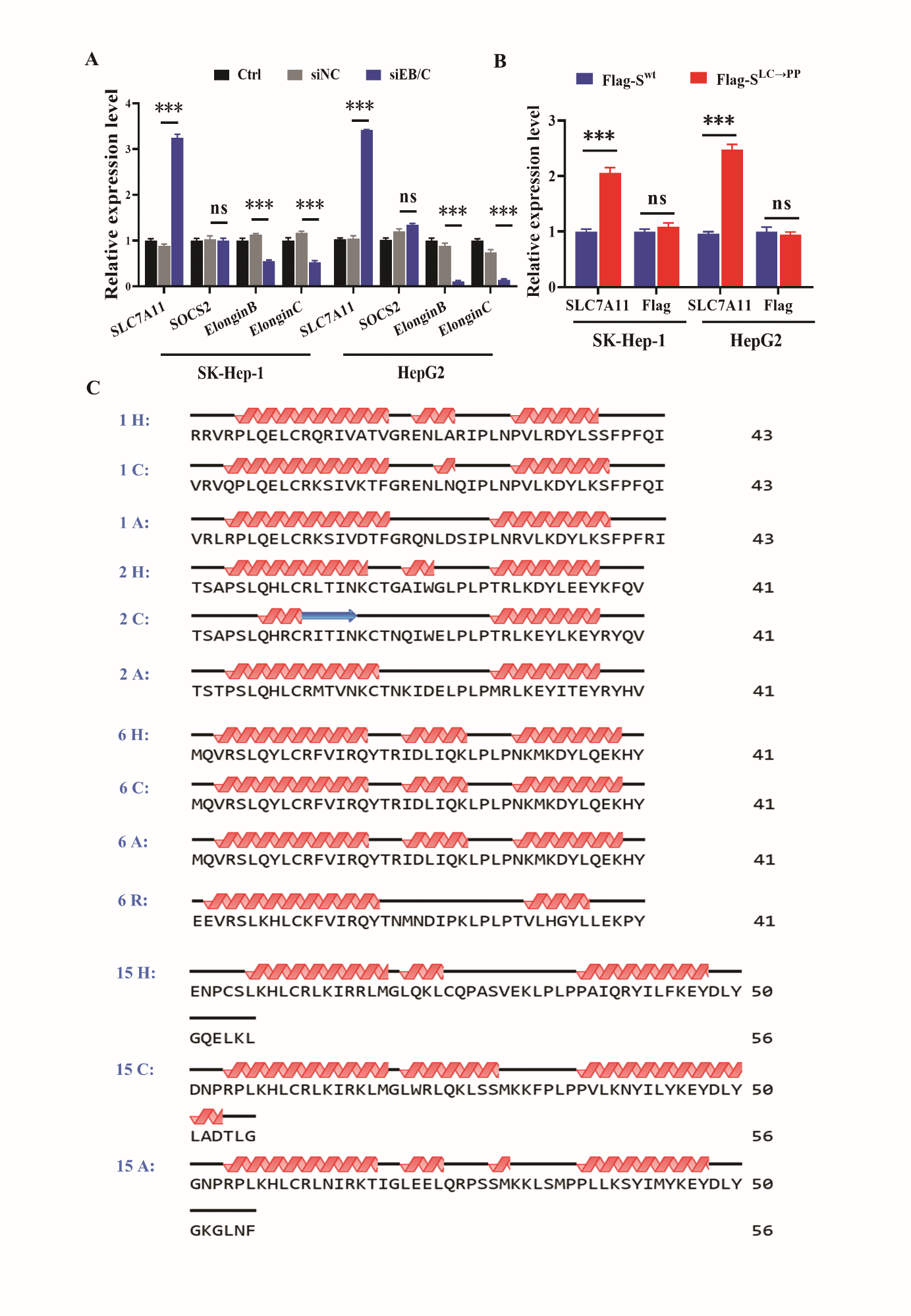
**

**Fig. S6**. Elongin B/C and SOCS2 co-promoted the ubiquitination degradation of SLC7A11. **(A)** Relative expression levels of SLC7A11, elongin B and elongin C proteins in the irradiated SK-Hep-1 and HepG2 cells transfected with siRNA targeting *elongin B* or *elongin C* (see Fig. 7B). **(B)** Relative expression levels of SLC7A11 and Flag proteins in the irradiated SK-Hep-1 and HepG2 cells transfected with SOCS2^WT^ or SOCS2^LC→PP^ plasmid (see Fig. 7F). Data were represented as mean ± SEM of at least three replicates. * *P* < 0.05, ** *P* < 0.01 and *** *P* < 0.001 between indicated groups. **(C)** Prediction of the secondary structure of SOCS2-BOX region in SOCS1, SOCS2, SOCS6 and SAB15 proteins from different species using PSIPRED tool. H refers to Human, C refers to Chicken, A refers to African clawed frog, R refers to Red flour beetle, and red wavy lines represent α-helix secondary structure.
