# Supplementary material for: SOCS2-enhanced ubiquitination of SLC7A11 promotes ferroptosis and radiosensitization in hepatocellular carcinoma
Source: Cell Death Differ. 2022 Aug 22;30(1):137–51. doi: 10.1038/s41418-022-01051-7 (PMC9883449; doi:10.1038/s41418-022-01051-7)
Supplement: Supplementary file 3 — Supplementary table 2 [file 41418_2022_1051_MOESM3_ESM.docx]

**Supplementary table S2. List of antibodies**

| **Antibody** | **Manufacturer** | **Catalog** | **Dilution** | **Assay** |
| --- | --- | --- | --- | --- |
| anti‐mouse IgG FITC | ThermoFisher (MA, USA) | F2761 | 1:200 | IF |
| anti‐rabbit IgG Cy3 | ThermoFisher (MA, USA) | A10520 | 1:200 | IF |
| anti‐mouse IgG Cy5 | ThermoFisher (MA, USA) | A10524 | 1:200 | IF |
| anti-mouse SOCS2 | Abnova Corporation (Shanghai, China) | H00008835-A01 | 1:1000  1:100  1:100 | WB  IF  Co-IP |
| anti-rabbit SOCS2 | Signalway Antibody  (MD, USA) | #49910 | 1:1000  1:100  1:100 | WB  IF  Co-IP |
| anti-mouse GPX4 | Santa Cruz Biotechnology (Shanghai, China) | sc-166570 | 1:1000  1:200  1:200  1:200 | WB  IF  IHC  Co-IP |
| anti-rabbit GPX4 | Abcam Technology (MA, USA) | ab125066 | 1:1000 | WB |
| anti-rabbit SLC7A11 | Cell signaling Technology  (TX, USA) | #12691S | 1:1000  1:200  1:200 | WB  IF  Co-IP |
| anti-mouse SLC7A11 | HuaBio, Inc. (Hangzhou, China) | HA600097 | 1:200 | IHC |
| anti-rabbit TCEB2/Elongin-B | Abcam Technology (MA, USA) | ab154854 | 1:1000 | WB |
| anti-rabbit TCEB1/Elongin-C | Proteintech Group  (Shanghai, China) | 12450-1-AP | 1:1000 | WB |
| anti-mouse Flag | Sigma-Aldrich (MO, USA) | F1804 | 1:1000 | WB |
| anti-rabbit Ub | Proteintech Group  (Shanghai, China) | 10201-2-AP | 1:1000 | WB |
| anti-mouse Ub | Cell signaling Technology  (TX, USA) | #3936S | 1:1000 | WB |
| anti-rabbit HA | Cell signaling Technology  (TX, USA) | #3724S | 1:1000 | WB |
| anti‐mouse IgG | Cell signaling Technology  (TX, USA) | #3420S | 1:100 | Co-IP |
| anti‐rabbit IgG | Cell signaling Technology  (TX, USA) | #3423S | 1:100 | Co-IP |
| anti-rabbit Tubulin | Proteintech Group  (Shanghai, China) | 11224-1-AP | 1:3000 | WB |
| anti-rabbit 4-HNE | Abcam Technology (MA, USA) | Ab46545 | 1:100 | IHC |
| anti-mouse Flag | Sigma-Aldrich (MO, USA) | F1804 | 1:1000  1:100 | WB  Co-IP |
