# Supplementary material for: SOCS2-enhanced ubiquitination of SLC7A11 promotes ferroptosis and radiosensitization in hepatocellular carcinoma
Source: Cell Death Differ. 2022 Aug 22;30(1):137–51. doi: 10.1038/s41418-022-01051-7 (PMC9883449; doi:10.1038/s41418-022-01051-7)
Supplement: Supplementary file 7 — Supplementary document [file 41418_2022_1051_MOESM7_ESM.pdf]

Sequence alignment results of SLC7A11 proteins in different species based on clustalw tool

Header line description

A:Sequence number B:Accession number(NP/XP) C:Amino acid number

D:Species E:Amino acid sequence

Underlined: Amino acid sequence of human-derived SLC7A11 protein

Yellow marker: 7 lysine sites susceptible to ubiquitination

A	B	C	D
Sequence 1:	NP_055146.1	501 aa	human
Sequence 2:	NP_036120.1	502 aa	house mouse
Sequence 3:	NP_001101143.2	502 aa	Norway rat
Sequence 4:	XP_009289503.1	498 aa	zebrafish
Sequence 5:	NP_001239111.1	491 aa	sheep
Sequence 6:	XP_021385250.1	503 aa	Bengalese finch
Sequence 7:	XP_024833346.1	503 aa	cattle
Sequence 8:	XP_002932094.2	500 aa	tropical clawed frog
Sequence 9:	XP_008265658.1	502 aa	rabbit
Sequence 10:	NP_001253220.1	501 aa	Rhesus monkey
Sequence 11:	NP_001288791.1	527 aa	dog
Sequence 12:	XP_001136486.1	501 aa	chimpanzee
Sequence 13:	XP_426289.3	501 aa	chicken
Sequence 14:	XP_035310922.1	502 aa	Chinese hamster
Sequence 15:	XP_005607903.1	503 aa	horse
Sequence 16:	XP_020957246.1	502 aa	pig
Sequence 17:	XP_044286854.1	503 aa	Komodo dragon
Sequence 18:	XP_039607615.1	498 aa	gray bichir
Sequence 19:	XP_044911795.1	489 aa	domestic cat
Sequence 20:	NP_001305270.1	503 aa	alpaca

B	E
NP_036120.1	MVRKPVVATISKGGYLQGNMSGRLPSMGDQEPPGQEKVVLKKKITLLRGVSIIIGTVIGS
NP_001101143.2	MVRKPVVATISKGGYLQGNVSGRLPSVGDQEPPGHEKVVLKKKITLLRGVSIIIGTVIGS
XP_035310922.1	MVRKPVVTTISKGGYLQGNVSGRLPSMGDQEAPGQEKVVLKKKITLLRGVSIIIGTVIGS
NP_055146.1	MVRKPVVSTISKGGYLQGNVNGRLPSLGNKEPPGQEKVQLKRKVTLLRGVSIIIGTIIGA
XP_001136486.1	MVRKPVVSTISKGGYLQGNVNGRLPSLGNKEPPGQEKVQLKRKVTLLRGVSIIIGTIIGA
NP_001253220.1	MVRKPVVSTISKGGYLQGNVNRRLPSLGNKEPPGQEKVQLKRKVTLLRGISIIIGTIIGA
NP_001288791.1	MVRKPVVSTISSGGYLQGNVNGKLPSLGGKEPPGQEKVVLKKKITLVRGISIIIGTIIGA
XP_044911795.1	MVRKPVVSTISSGGYLQGNVNGKLPSLGGKEPPGQEKVVLKKKITLLRGISIIIGTIIGA
XP_005607903.1	MVRKPVVSTISNGGYLQGNVNGRLPSLGSKEPPEQEKVVLKKKITLLRGISIIIGTIIGA
NP_001239111.1	MVRKPVVSTISNGAYLQGSVNGRLPAPGGKEPPGQEKVVLKKKITLLRGISIIIGTIIGA
XP_024833346.1	MVRKPVVSTISSGAYLQGSVNGRLPALGGREPPGQEKVVLKKKITLLRGISIIIGTIIGA
XP_020957246.1	MVRKPVVSTISNGGYLQGNVHRRLPSLGGREPPGQEKVVLKKKITLLRGISIIIGTIIGA
NP_001305270.1	MVRKPVVSTISNGGYLQGNVHGRLPSLGSKEPPGQERVVLKKKITLLRGISIIIGTIIGA
XP_008265658.1	MVRKPVVSTISKRSYLQGNVNGRLPSMGGKEPPGQEKVVLKKKITLLRGISIIIGTIIGA
XP_021385250.1	MFRKAAVPTVSNGSYLQGRANGKLSSMDSGQPAREGKVVLKKKVTLLRGISIIIGTIIGA
XP_426289.3	MVRKAAVVTVPNGSYLQGQPNGRLPSMDSRQPQE--KVVLKKKVTLLRGISIIIGTIIGA

```
XP_044286854.1      MFRKPPASAISNGSYLQGQSNGKLSAVRDTKAAQEDKVVLKKKVTLLRGISIIIGTIIGA
XP_002932094.2      --MYRQAADDVYSVTKGKDSRRMLPSADKKMSEKGEKVVLKKKVTLLRGISIIIGTIIGA
XP_009289503.1      MPRRTVSAPHPNGDPTPSN-FGEKEPLKLNGEPPKEKVELRKKVTLLRGISIIIGTIIGA
XP_039607615.1      MGRKPVNPSISNGGYAKRDSSGQFDISDCNNTTVSDKVELKKKVTLLRGISLITGTIIGA
                                                        :* *::*:**:**:*:* **:**:

NP_036120.1         GIFISPKGILQNTGSVGMSLVFWSACGVLSLFGALSYAELGTSIKKSGGHYTYILEVFGP
NP_001101143.2      GIFISPKGILQNTGSVGMSLVFWSACGVLSLFGALSYAELGTSIKKSGGHYTYILEVFGP
XP_035310922.1      GIFISPKGILQNTGSVGMSLVFWSACGVLSLFGALSYAELGTSIKKSGGHYTYILEVFGP
NP_055146.1         GIFISPKGVLQNTGSVGMSLTIWTVCGVLSLFGALSYAELGTTIKKSGGHYTYILEVFGP
XP_001136486.1      GIFISPKGVLQNTGSVGMSLTIWTVCGVLSLFGALSYAELGTTIKKSGGHYTYILEVFGP
NP_001253220.1      GIFISPKGVLQNTGSVGMSLTIWTVCGVLSLFGALSYAELGTSIKKSGGHYTYILEVFGP
NP_001288791.1      GIFISPKGVLQNTGSVGMSLVIWTVCGVLSLFGALSYAELGTSIKKSGGHYTYILEVFGP
XP_044911795.1      GIFISPKGVLQNTGSVGMSLIIWTVCGVLSLFGALSYAELGTSIKKSGGHYTYILEVFGP
XP_005607903.1      GIFISPKGVLQNTGSVGMSLIIWTVCGVLSLFGALSYAELGTSIKKSGGHYTYILEVFGP
NP_001239111.1      GIFISPKGVLQNTGSVGLSLVVWSVCGVLSLFGALSYAELGTSIKKSGGHYTYILEVFGP
XP_024833346.1      GIFISPKGVLQNTGSVGLSLVVWTVCGVLSLFGALSYAELGTSIKKSGGHYTYILEVFGP
XP_020957246.1      GIFISPKGVLQNTGSVGLSLVVWTVCGVLSLFGALSYAELGTSIKKSGGHYTYILEVFGP
NP_001305270.1      GIFISPKGILQNTGSVGLSLVIWTVCGVLSLFGALSYAELGTSIKKSGGHYTYILEVFGP
XP_008265658.1      GIFISPKGVLRNTGTVGMSLVVWTLCGVLSLFGALSYAELGTSIKKSGGHYTYILEVFGP
XP_021385250.1      GIFISPKGILKNTGSVGMSLTVWTVCGILSLFGALCYAELGTCIKKSGGHYTYILEAFGP
XP_426289.3         GIFISPKGILKNTGSVGMSLIVWTACGILSLFGALCYAELGTCIKKSGGHYTYILEAFGP
XP_044286854.1      GIFISPKGILQHTGSVGMSLTVWTVCGILSLFGALCYAELGTSIKKSGGHYTYILEAFGP
XP_002932094.2      GIFISPKGILQNTGSVGMSLVVWTACGILSLFGALCYAELGTCIKKSGGHYTYILEAFGP
XP_009289503.1      GIFISPKGILQNSGSVGMSLVVWVACGILSLFGALSYAELGTCIKKSGGHYTYILEAFGP
XP_039607615.1      GIFISPKGILKNSGSVGMSLIIWTACGVLSLFGALSYAELGTCIKKSGGHYTYILEAFGP
                    ********:*:::*:**:** .*  **:*******.****** *************.***

NP_036120.1         LLAFVRVWVELLVIRPGATAVISLAFGRYILEPFFIQCEIPELAIKLVTAVGITVVMVLN
NP_001101143.2      LLAFVRVWVELLVIRPGATAVISLAFGRYILEPFFIQCEIPELAIKLVTAVGITVVMVLN
XP_035310922.1      LLAFVRVWVELLIIRPGATAVISLAFGRYILEPFFIQCEIPELAIKLVTAVGITVVMVLN
NP_055146.1         LPAFVRVWVELLIIRPAATAVISLAFGRYILEPFFIQCEIPELAIKLITAVGITVVMVLN
XP_001136486.1      LPAFVRVWVELLIIRPAATAVISLAFGRYILEPFFIQCEIPELAIKLITAVGITVVMVLN
NP_001253220.1      LPAFVRVWVELLIIRPAATAVISLAFGRYILEPFFIQCEIPELAIKLITAVGLTVVMVLN
NP_001288791.1      LPAFVRVWVELLIIRPAATAVISLAFGRYILEPFFIQCEIPELAIKLITAVGITVVMVLN
XP_044911795.1      LPAFVRVWVELLIIRPAATAVISLAFGRYILEPFFIQCEIPELAIKLITAVGITVVMVLN
XP_005607903.1      LPAFVRVWVELLIIRPAATAVISLAFGRYILEPFFIQCEIPELAIKLITAVGITVVMVLN
NP_001239111.1      LPAFVRVWVELLIIR------------HMMEQQFSPLEDLRRPQYWLPRCLLSSVVMVLN
XP_024833346.1      LPAFVRVWVELLIIRPAATAVISLAFGRYILEPFFIHCEIPELAIKLITAVGITVVMVLN
XP_020957246.1      LPAFVRVWVELLIIRPAATAVISLAFGRYILEPFFIQCEIPELAIKLITAVGITVVMVLN
NP_001305270.1      LPAFVRVWVELLIIRPAATAVISLAFGRHILEPFFIQCEIPELAIKLITAVGITVVMVLN
XP_008265658.1      LLAFVRVWVELLIIRPAATAVISLAFGRYILEPFFIQCEIPELAIKLVTAVGITAVMVLN
XP_021385250.1      LPAFVRVWVELLIIRPAATAVISLAFGRYILEPFFMQCEIPELAVKLITAVGITLVMVLN
XP_426289.3         LPAFVRVWVELLVIRPAATAVISLAFGRYILEPFFMQCEIPELAIKLITAVGITLVMVLN
XP_044286854.1      LPAFIRVWVELLIIRPAATAVISLAFGRYILEPFFMQCEIPVLAVKLVTAVGITIVMVLN
XP_002932094.2      LPAFIRVWVELLVIRPAATAVISLAFGRYILEPFFIQCEIPDLAIKLVTALGITIVMVLN
XP_009289503.1      QVAFVRLWADMIAIRPAGLAVIALAFGRYILEPIFMPCGVPEIAIKLATTIGITIVMYLN
XP_039607615.1      LAAFVRLWVEVLVIRPAATAVISLAFGQYILQPLFMPCEVPELLMKIVTSIGITSVMYLN
                      **:*:*.::: **            : : : :     :      :   :  : ** **
```

```
NP_036120.1             STSVSWSARIQIFLTFCKLTAILIIIVPGVIQLIKGQTHHFKDAFSGRDTSLMGLPLAFY
NP_001101143.2          STSVSWSARIQIFLTFCKLTAILIIIVPGVIQLIKGQTHHFKDAFSGRDTNLMGLPLAFY
XP_035310922.1          SMSVSWSARIQIFLTFCKLTAILIIIVPGVIQLIKGHTQHFEDAFSGRDANLMGLPLAFY
NP_055146.1             SMSVSWSARIQIFLTFCKLTAILIIIVPGVMQLIKGQTQNFKDAFSGRDSSITRLPLAFY
XP_001136486.1          SMSVSWSARIQIFLTFCKLTAILIIIVPGVMQLIKGQTQNFKDAFSGRDSSITRLPLAFY
NP_001253220.1          SMSVSWSARIQIFLTFCKLTAILIIIVPGVMQLIKGQTQNFKDAFSGRDSSITRLPLAFY
NP_001288791.1          SMSVSWSARIQIFLTFCKLTAILIIIVPGVMQLIKGQTQHFKDAFSGRGANIMGLPLAFY
XP_044911795.1          SMSVSWSARIQIFLTFCKLTAILIIIVPGVMQLIKGQTQHFQDAFSGRDASIMGLPLAFY
XP_005607903.1          SMSVSWSARIQIFLTFCKLTAIVIIIVPGVMQLIKGQTQHFKDAFSGRDANIMGLPLAFY
NP_001239111.1          SMSVSWSARIQIFLTFCKLTAILIIIVPGVMQLIKGKHSILKMPFQEEMQVLWGCHWLFI
XP_024833346.1          SMSVSWSARIQIFLTFCKLTAILIIIVPGVMQLIKGQTQYFKDAFSGRDASIMGLPLAFY
XP_020957246.1          SMSVSWSARIQIFLTFCKLTAILIIIVPGVIQLIKGQTQHFKDAFSGRNESIMGLPLAFY
NP_001305270.1          SMSVSWSARIQIFLTFCKLTAILIIIVPGVMQLIKGQTQHFKDAFSGRDASIMGLPLAFY
XP_008265658.1          SMSVSWSARIQIFLTFCKLTAILIIIVPGVMQLIKGQTQHFKDAFSGRDASVMGLPLAFY
XP_021385250.1          STSVSWSARIQMFLTFCKLVAILIIIVPGVIQLIKGETQHFKNAFAGNDASIMGLPLAFY
XP_426289.3             STSVSWSARIQIFLTFCKLVAILIIIVPGVIQLIKGETQHFKNAFSGNDASIMGLPLAFY
XP_044286854.1          SMSVSWSTRIQIFLTFCKLIAILIIIVPGVMQLIKGENQHFKDAFVGNAASVKGLPLAFY
XP_002932094.2          SVSVSWSARIQIFLTFCKLIAILIIIVPGVLQLIKGETQNFKDAFIGKDVSVMGLPLAFY
XP_009289503.1          SMSVSWTARLQIFLTFSKLLAIAIIIVPGLYQLFKGETKNFENAFEVNTAQLTGLPLAFY
XP_039607615.1          SMSVSWSARIQIFLTFCKLLAMLIIIFPGLFQLFKGETQNFHNAFGVNEVPVMGLPLAFY
                        * ****::*:*:****.** *: ***.**: **:**.   :. .*  .   :      *

NP_036120.1             YGMYAYAGWFYLNFITEEVDNPEKTIPLAICISMAIITVGYVLTNVAYFTTISAEELLQS
NP_001101143.2          YGMYAYAGWFYLNFITEEVDNPEKTIPLAICISMAIITVGYVLTNVAYFTTISAEELLQS
XP_035310922.1          YGMYAYAGWFYLNFITEEVENPEKNIPLAICISMAIITVGYVLTNVAYFTTISAEELLLS
NP_055146.1             YGMYAYAGWFYLNFVTEEVENPEKTIPLAICISMAIVTIGYVLTNVAYFTTINAEELLLS
XP_001136486.1          YGMYAYAGWFYLNFVTEEVENPEKTIPLAICISMAIVTIGYVLTNVAYFTTINAEELLLS
NP_001253220.1          YGMYAYAGWFYLNFVTEEVENPEKTIPLAICISMAIVTIGYVLTNVAYFTTINAEELLLS
NP_001288791.1          YGMYAYAGWFYLNFVTEEVENPEKTIPLAICISMAIVTIGYVLTNVAYFTTISAEELLLS
XP_044911795.1          YGMYAYAGWFYLNFVTEEVENPEKTIPLAICISMAIVTTGYVLTNVAYFTTISAEELLLS
XP_005607903.1          YGMYAYAGWFYLNFLTEEVENPEKTIPLAICISMAIVTIGYVLTNVAYFTTISAEELLLS
NP_001239111.1          MECMRTPAGFYLNFVTEEVENPEKTIPLAICISMTVVTVGYVLTNVAYFTTISAEELMLS
XP_024833346.1          YGMYAYAGWFYLNFVTEEVENPEKTIPLAICISMAIVTVGYVLTNVAYFTTISAEELMLS
XP_020957246.1          YGMYAYAGWFYLNFVTEEVENPEKTIPLAICISMAIVTTGYVLTNVAYFTTISAEELLLS
NP_001305270.1          YGMYAYAGWFYLNFVTEEVENPEKTIPLAICISMAIVTVGYVLTNVAYFTTISAEELLLS
XP_008265658.1          YGMYAYAGWFYLNFVTEEVENPERTVPLAICISMAIVTIGYVLTNVAYFTTISAEELLLS
XP_021385250.1          SGMYAYSGWFYLNFVTEEVENPEKNIPLAICISMIIVTVGYVLTNVAYFTTISAGELLLS
XP_426289.3             SGMYAYSGWFYLNFVTEEVENPEKNIPLAICISMIIVTVGYVLTNVAYFTTISAGELLLS
XP_044286854.1          SGMYAYSGWFYLNFVTEEVENPERNVPLAICTSMVIVTVGYVLTNVAYFTTISPEELLMS
XP_002932094.2          SGMYAYAGWFYLNFVTEEVENPEKNVPLAICISMAIVTVGYVLTNVAYYTTISAEELVLS
XP_009289503.1          SGMYAYAGWFYLNFVTEEVENPERTVPLAICISMVIVMICYTLTNVAYYTVMSADELLAS
XP_039607615.1          SGMYAYAGWFYLNFVTEEVENPEKTVPLAICLSMVIVTFSYVLINVAYYAVLTADEMLAS
                              .. *****:****:***:.:***** ** ::   *.* ****::.:.. *:: *

NP_036120.1             SAVAVTFSERLLGKFSLAVPIFVALSCFGSMNGGVFAVSRLFYVASREGHLPEILSMIHV
NP_001101143.2          SAVAVTFSERLLGKFSLAVPIFVALSCFGSMNGGVFAVSRLFYVASREGHLPEILSMIHV
XP_035310922.1          NAVAVTFSERLLGNFSLAVPIFVALSCFGSMNGGVFAVSRLFYVASREGHLPEILSMIHV
NP_055146.1             NAVAVTFSERLLGNFSLAVPIFVALSCFGSMNGGVFAVSRLFYVASREGHLPEILSMIHV
XP_001136486.1          NAVAVTFSERLLGNFSLAVPIFVALSCFGSMNGGVFAVSRLFYVASREGHLPEILSMIHV
NP_001253220.1          NAVAVTFSERLLGNFSLAVPIFVALSCFGSMNGGVFAVSRLFYVASREGHLPEILSMIHV
```

```
NP_001288791.1     NAVAVTFSERLLGNFSLAVPIFVALSCFGSMNGGVFAVSRLFYVASREGQLPEILSMIHV
XP_044911795.1     NAVAVTFSERLLGNFSLAVPIFVALSCFGSMNGGVFAVSRLFYVASREGHLPEILSMIHV
XP_005607903.1     NAVAVTFSERLLGNFSLAVPIFVALSCFGSMNGGVFAVSRLFYVASREGHLPEILSMIHV
NP_001239111.1     NAVAVTFSERLLGNFSLAVPIFVALSCFGSMNGGVFAVSRLFYVASREGHLPEILSMIHI
XP_024833346.1     NAVAVTFSERLLGNFSLAVPIFVALSCFGSMNGGVFAVSRLFYVASREGHLPEILSMIHI
XP_020957246.1     KAVAVTFSERLLGNFSLAVPIFVALSCFGSMNGGVFAVSRLFYVASREGHLPEILSMIHI
NP_001305270.1     SAVAVTFSERLLGNLSLAVPIFVALSCFGSMNGGVFAVSRLFYVASREGHLPEILSMIHV
XP_008265658.1     NAVAVTFSERLLGNFSLAVPIFVALSCFGSMNGGVFAVSRLFYVASREGHLPEILSMIHV
XP_021385250.1     KAVAVTFAERLMGNFSLAVPVFVALSCFGSMNGGIFAVSRMFFVASREGHLPEILSMIHV
XP_426289.3        KAVAVTFAERLMGSFSLAVPVFVALSCFGSMNGGIFAVSRMFFVASREGHLPEILSMIHV
XP_044286854.1     KAVAVTFAERLLGSFSLAVPIFVALSCFGSMNGGVFAVSRMFYVASREGHLPEILSMIHV
XP_002932094.2     SAVAVTYAERLFGSFSLAVPIFVALSCFGSMNGGVFAVSRMFYVASREGHLPEILSMIHV
XP_009289503.1     NAVAVTFAEKLMGNFSYAVPVFVALSCFGSMNGCLFAISRMFFVASREGQLPEVLSMIHI
XP_039607615.1     DAVAMSFATKLMGKFSIAVPIFVSLSCYGSMNGGIFAVSRMFYAASREGHLPELLSMVHI
                   .***:::: :*:*.:* ***:**:***:***** :**:**:*:.*****:***:***:*:

NP_036120.1        HKHTPLPAVIVLHPLTMVMLFSGDLYSLLNFLSFARWLFMGLAVAGLIYLRYKRPDMHRP
NP_001101143.2     HKHTPLPAVIVLHPLTMIMLFSGDLYSLLNFLSFARWLFMGLAVAGLIYLRYKRPDMHRP
XP_035310922.1     HKHTPLPAVIVLHPLTMIMLFSGDLYSLLNFLSFARWLFMGLAVAGLIYLRYKRPDMHRP
NP_055146.1        RKHTPLPAVIVLHPLTMIMLFSGDLDSLLNFLSFARWLFIGLAVAGLIYLRYKCPDMHRP
XP_001136486.1     RKHTPLPAVIVLHPLTMIMLFSGDLDSLLNFLSFARWLFIGLAVAGLIYLRYKCPDMHRP
NP_001253220.1     RKHTPLPAVIVLHPLTMIMLFSGDLDSLLNFLSFARWLFIGLAVAGLIYLRYKCPDMHRP
NP_001288791.1     RKHTPLPAVIVLHPLTMIMLFSGDLYSLLNFLSFARWLFIGLAVAGLIYLRYKRPDMHRP
XP_044911795.1     RKHTPLPAVIVLHPLTMIMLFSGDLYSLLNFLSFARWLFIGLAVAGLIYLRYKRPEMHRP
XP_005607903.1     RKHTPLPAVIVLHPLTMIMLFSGDLYSLLNFLSFARWLFIGLAVAGLIYLRYKRPDMHRP
NP_001239111.1     RKHTPLPAVIVLHPLTMIMLFSGDLYSLLNFLSFARWLFIGLAVAGLIYLRYKRPDMHRP
XP_024833346.1     RKHTPLPAVIVLHPLTMIMLFSGDLYSLLNFLSFARWLFIGLAVAGLIYLRYKRPDMHRP
XP_020957246.1     RKHTPLPAVIVLHPLTMIMLFSGDLYSLLNFLSFARWLFIGLAVAGLIYLRYKRPDMHRP
NP_001305270.1     RKHTPLPAVIVLHPLTMIMLFSGDLYSLLNFLSFARWLFIGLAVAGLIYLRYKRPDMHRP
XP_008265658.1     RKHTPLPAVIVLHPLTMIMLFSGDLYSLLNFLSFXRWLFIGLAVAGLIYLRYKRPDMQRP
XP_021385250.1     RKHTPLPAVIVLHPLTMIMLFNGDLYSLLNFLSFARWLFIGLVVAGLIYLRYKRPDMPRP
XP_426289.3        RKHTPLPAVIVLHPLTMIMLFSGDLYSLLNFLSFARWLFIGLVVAGLIYLRYKRPDMPRP
XP_044286854.1     RKHTPLPAVIVLYPLTMIMLFTSDLYSLLNFLSFARWLFIGLAVAGMIYLRYKRPDMPRP
XP_002932094.2     RKHTPLPAVIVLLPLTMVMLFTGDIYSLLNFFSFVRWLFIGLAVAGLIYLRYKRPEMHRP
XP_009289503.1     RRHTPLPAVIVLYPITLLILFLGDIYSLLNFMSFLRWLFIGVAVVGLIYLRFTRPDLPRP
XP_039607615.1     RKHTPLPAVIFLYPLTILMLFVGDIYSLLNFMSFVRWLFIGIVIVGLIYLRYKQPQMHRP
                   ::********.* *:*:::** .*: *****:** ****:*:.:.*:****:. *:: **

NP_036120.1        FKVPLFIPALFSFTCLFMVVLSLYSDPFSTGVGFLITLTGVPAYYLFIVWDKKPKWFRRL
NP_001101143.2     FKVPLFIPALFSFTCLFMVVLSLYSDPFSTGVGFLITLTGVPAYYLFIVWDKKPKWFRRL
XP_035310922.1     FKVPLFIPALFSFTCLFMVVLSLYSDPFSTGVGFLITLTGVPAYYLFIVWDKKPKWFRQL
NP_055146.1        FKVPLFIPALFSFTCLFMVALSLYSDPFSTGIGFVITLTGVPAYYLFIIWDKKPRWFRIM
XP_001136486.1     FKVPLFIPALFSFTCLFMVALSLYSDPFSTGIGFVITLTGVPAYYLFIIWDKKPRWFRIM
NP_001253220.1     FKVPLFIPALFSFTCLFMVALSLYSDPFSTGIGFIITLTGVPAYYLFIIWDKKPKWFRIM
NP_001288791.1     FKVPLFIPALFSFTCLFMVALSLYSDPFSTGIGFIITLTGVPAYYLFIIWDKKPKWFRRL
XP_044911795.1     FKVPLFIPALFSFTCLFMVALSLYSDPFSTGIGFIITLTGVPAYYLFIIWDKKPKWFRRM
XP_005607903.1     FKVPLFIPALFSFTCLFMVALSLYSDPFSTGIGFVITLTGVPAYYLFIIWDKKPRWFRRM
NP_001239111.1     FKVPLFIPALFSFTCLFMVALSLYSDPFSTGIGFIITLTGIPAYYLFIIWDKKPKWFRRM
XP_024833346.1     FKVPLFIPALFSFTCLFMVALSLYSDPFSTGIGFIITLTGIPAYYLFIIWDKKPKWFRRM
XP_020957246.1     FKVPIFIPALFSFTCLFVVALSLYSDPFSTGIGFIITLTGVPAYYLFIIWDKKPKWFRRM
```

```
NP_001305270.1      FKVPLFIPALFSFTCLFMVALSLYSDPFSTGIGFIITLTGVPAYYLFIIWDKKPKWFRRM
XP_008265658.1      FKVPLFIPALFSFTCFFMVALSLYSDPFSTGIGFVITLSGVPAYYLFIIWDKKPKWFRRM
XP_021385250.1      FKVPLFIPALFSFTCLFMVALSLYSDPVNTGIGFAITLTGIPAYYFFIVWDNKPKWFRKL
XP_426289.3         FKVPLFIPALFSFTCLFMVALSLYSDPVNTGIGFAITLTGVPAYYLFIVWDKKPKWFRKL
XP_044286854.1      FKVPLFIPALFSFTCLFMVVLSLYADPVNTGIGFAITLTGVPAYYLFIIWDKKPTWFRRF
XP_002932094.2      FKVPIFIPALFSFTCLFMVALSLYSDPINTGIGFAITLTGVPAYYLFVIWDNKPEWFRTF
XP_009289503.1      FKVPIFIPAVFSFTCFFMVFLSLYSDPINTGIGFAISLTGIPAYYIFIHSKRKPKWFQKF
XP_039607615.1      FKVPLFIPALFCFSCFLMVGLSLYSDPINTGIGFGITLTGIPAYYIFIVWNKKPKIIQKF
                    ****:****:*.*:*:::* ****:**..**:** *:*:*:****:*:  ..**  :: :

NP_036120.1         SDRITRTLQIILEVVPEDSKEL-------------------------
NP_001101143.2      SDRITRTLQIILEVVPEDSKEL-------------------------
XP_035310922.1      SERITRTLQIILEVVPEDSKEL-------------------------
NP_055146.1         SEKITRTLQIILEVVPEEDKL--------------------------
XP_001136486.1      SEKITRTLQIILEVVPEEDKL--------------------------
NP_001253220.1      SERITRTLQIILEVVPEEDKL--------------------------
NP_001288791.1      SGKTFTQFWDLLRDRVIENPVYGYTQPHTHTHTHPLMFNNVSKATAH
XP_044911795.1      SERTTSNDP--------------------------------------
XP_005607903.1      SDRITRTLQIILEVVPEEDCHKL------------------------
NP_001239111.1      SDIITRTLQIILEVVPEEDCHKL------------------------
XP_024833346.1      SDMITRTLQIILEVVPEEDCHKL------------------------
XP_020957246.1      SDRITRTLQIILEVVPEEDCHK-------------------------
NP_001305270.1      SDKITRTLQIILEVVPEEDCQKL------------------------
XP_008265658.1      SDTITRTLQIILEVVPEEDCRK-------------------------
XP_021385250.1      LGRVTTTLQILLEVIPAEEDQKS------------------------
XP_426289.3         LDTVTEALQILLEVVPSEEDQKS------------------------
XP_044286854.1      LGRITSMLQIILEVVPAEENPRS------------------------
XP_002932094.2      TDRTTLMLQILLEVAPTDKNIS-------------------------
XP_009289503.1      SDSMNRSLQILLEVVPAEH----------------------------
XP_039607615.1      TDSTTRTLQIILEVVSPE-----------------------------
```
